# Observational and genetically-informed characterization of Gulf War Illness symptoms in 1990–1991 Gulf War deployed Veterans and non-deployed Gulf War Era Veterans

**DOI:** 10.1186/s12940-026-01316-5

**Published:** 2026-06-11

**Authors:** Jun He, Gita A. Pathak, Brenda Cabrera-Mendoza, Dan Qiu, Kelson Zawack, Lea Steele, Rachel Quaden, Kelly M. Harrington, Elizabeth J. Gifford, Mihaela Aslan, Drew A. Helmer, Elizabeth R. Hauser, Renato Polimanti

**Affiliations:** 1 Cooperative Studies Program Clinical Epidemiology Research Center (CSP-CERC), VA Connecticut Healthcare System, West Haven, CT 06516 USA; 2 Department of Psychiatry, Yale University School of Medicine, 60 Temple St, Suite 7A, New Haven, CT 06510 USA; 3 Department of Internal Medicine, Yale University School of Medicine, New Haven, CT 06511 USA; 4 Veterans Health Research Program, Yudofsky Division of Neuropsychiatry, Department of Psychiatry and Behavioral Sciences, Baylor College of Medicine, Houston, TX 77030 USA; 5 Million Veteran Program (MVP) Coordinating Center, VA Boston Healthcare System, Boston, MA 02130 USA; 6 Department of Psychiatry, Boston University Chobanian & Avedisian School of Medicine, Boston, MA 02118 USA; 7 Center for Innovations in Quality, Effectiveness, and Safety (IQuESt), Michael E. DeBakey VA Medical Center, Houston, TX 77030 USA; 8 Department of Medicine, Baylor College of Medicine, Houston, TX 77030 USA; 9 VA Cooperative Studies Program Epidemiology Center-Durham, Department of Veterans Affairs, Durham, NC 27705 USA; 10 Center for Child and Family Policy, Duke Margolis Center for Health Policy, Duke University Sanford School of Public Policy, Durham, NC 27708 USA; 11 Department of Biostatistics and Bioinformatics, Duke Molecular Physiology Institute, Duke University, Durham, NC 27705 USA; 12 Department of Biomedical Informatics and Data Science, Yale School of Medicine, New Haven, CT 06511 USA; 13 Department of Chronic Disease Epidemiology, Yale School of Public Health, New Haven, CT 06511 USA; 14 Wu Tsai Institute, Yale University, New Haven, CT 06511 USA

**Keywords:** Gulf War Illness, Symptoms, Deployment, Genetic liability, Veterans

## Abstract

**Background:**

Gulf War Illness (GWI) has been identified in more than 20% of Veterans following military service in the 1990–1991 Gulf War (GW). Unfortunately, after several decades, GWI pathogenesis remains unclear and the patterns of GWI symptom co-occurrence are still poorly understood.

**Methods:**

This population-based case–control study included 34,179 (16% females; 34% GW deployed; mean age 61 years) Veterans enrolled in the Million Veteran Program (MVP) who were active-duty military personnel in 1990–1991. We analyzed 14 GWI symptoms in GW-deployed and non-deployed GW-Era Veterans. Multivariable generalized linear models were used to estimate GWI symptom associations with demographic factors and deployment status. Correlation, factor, and network analyses were performed to examine the underlying structure of GWI symptoms. In addition, polygenic risk score and one-sample Mendelian randomization analyses were conducted to evaluate the genetically inferred effects of health-related traits on symptom factors.

**Results:**

Among 14 GWI symptoms, joint pain had the highest prevalence in both GW deployed (86%) and non-deployed GW-Era Veterans (79%). The median age of symptom onset ranged from 38 to 46 years among deployed Veterans and from 45 to 54 years among non-deployed Veterans. Factor analysis identified a five-factor latent structure as the best-fit model for GWI symptoms, and latent class analysis classified Veterans into seven symptom classes. The network of GWI symptoms showed that fatigue had the largest closeness and betweenness and had more edges in deployed Veterans. Younger age, female sex, non-European descent, lower educational attainment, enlisted rank, and GW deployment, as well as multiple GW-related exposures in deployed Veterans, were associated with increased GWI symptom burden. Genetic liability to multiple health-related outcomes, including type 2 diabetes (T2D) and posttraumatic stress disorder (PTSD), showed putative causal effects on GWI-symptom factors, with the T2D effect being larger in deployed Veterans and the PTSD effect being larger in non-deployed Veterans.

**Conclusions:**

The present findings demonstrate differences in symptom onset between deployed and non-deployed GW Veterans. They also reinforce the importance of symptom clusters of relevance to GWI, associations between deployment-specific exposures and symptom burden, and different genetic linkages underlying symptoms for deployed and non-deployed groups.

**Trial registration:**

Not applicable.

**Supplementary Information:**

The online version contains supplementary material available at 10.1186/s12940-026-01316-5.

## Background

Gulf War Illness (GWI) is a multi-symptom, chronic condition affecting Veterans who deployed to the Persian Gulf region during Operations Desert Storm and Desert Shield in 1990–1991 [1]. These symptoms span multiple domains and systems, such as musculoskeletal, gastrointestinal, mood, and cognitive issues [2]. Currently, two primary research case definitions for GWI are widely used: the U.S. Centers for Disease Control and Prevention (CDC) definition [3] and the Kansas definition [4]. In October 2025, GWI was formally recognized in the International Classification of Diseases, 10th Revision, Clinical Modification (ICD-10-CM), with diagnostic criteria adapted from the Kansas definition [5, 6]. Although the prevalence of GWI varies depending on the diagnostic criteria applied, it is estimated that among the nearly 700 000 Veterans deployed to the Gulf War (GW) theater, approximately 175 000–210 000 (~ 30%) are afflicted [2]. GWI has been associated with serious health outcomes, including cardiometabolic diseases, sleep disturbance, and posttraumatic stress disorder (PTSD) [7, 8], significantly impairing the quality of life of affected Veterans and imposing a substantial disease burden on society [8].

The etiology of GWI has not been fully elucidated. Multiple reports indicate that troops encountered a broad range of potentially hazardous exposures during GW deployment. Studies have most consistently identified the use of pyridostigmine bromide (PB) pills (prophylaxis against nerve agent effects) and pesticide exposures during GW deployment as significant risk factors for GWI [2, 9–11]. Other GW-related exposures, such as nerve agents, smoke from over 600 burning oil well fires, and vaccines, may also have contributed to GWI [2, 9, 12–14]. In addition, GWI has been associated with multiple biological alterations, including changes in central nervous system structure, lipid abnormalities, and immune function [2, 15–18].

Our genome-wide association study (GWAS) of GWI expanded the understanding of the disease etiology by assessing case–control status considering CDC and Kansas criteria [19]. Specifically, we considered CDC and Kansas GWI-case definitions, applying their specific strategies to distinguish symptoms due to GWI from those attributable to unrelated conditions (severity threshold and exclusionary criteria, respectively). Additionally, we investigated less stringent definitions, considering symptoms included in CDC and Kansas criteria without applying severity threshold and exclusionary criteria, respectively. Our analyses highlighted that while there are similar genetic correlations across these GWI-case definitions, there are also important differences. First, without applying a severity threshold and exclusionary criteria, the majority of Veterans in our cohort (> 65%) are defined as GWI cases considering symptoms included in both CDC and Kansas definitions. Conversely, applying the complete CDC and Kansas definitions (symptoms plus severity threshold or exclusionary criteria, respectively), GWI prevalence in our sample was in line with what reported in other GWI studies (25–30%) [2]. However, compared to the assignment of the CDC severity threshold, applying the Kansas exclusionary criteria demonstrated markedly fewer genetic findings (i.e., null heritability estimates and fewer polygenic risk associations) [19]. Also, by removing Veterans with exclusionary conditions from analyses, Veterans who developed GWI prior to development of the exclusionary conditions were not identified as GWI cases, and the generalizability and clinical relevance of the findings of genetic association were reduced. Accordingly, our findings supported the use of CDC-severe case definition for future genetic studies of GWI.

Previous studies have estimated the prevalence of GWI symptoms and linked them to deployment status and other factors [20, 21], but few of them have examined the causal relationships using genetically informed methods [22, 23]. Furthermore, limited evidence exists on the extent to which genetic and environmental factors contribute to their manifestation [24]. Accordingly, characterizing symptom patterns across different deployment subgroups and evaluating the long-term effects of GW deployment may enhance our understanding of GWI.

In this study, leveraging data from the U.S. Department of Veterans Affairs (VA) Million Veteran Program (MVP), we investigated the associations of demographic factors and deployment status with GWI symptoms, identified the underlying structure and network of GWI symptoms, and explored genetically informed causal relationships with other health-related outcomes. By integrating phenotypic and genetically informed approaches in GW deployed Veterans (i.e., Veterans who served in the Gulf War theater; hereafter referred to as deployed Veterans) and non-deployed GW-Era Veterans (i.e., Veterans who were in the military during the 1990–1991 GW period but did not serve in the Gulf War theater; hereafter referred to as non-deployed Veterans), we aimed to deconstruct the interrelationships of GWI symptoms and identify their external influencing factors, thereby providing new insights into GWI etiology, pathobiology, and GWI prevention and intervention strategies.

## Methods

### Study design and participants

This study utilized data from the 1990–1991 GW-Era Survey conducted under the MVP 029/CSP #2006 project [20, 25]. Veterans who served in the U.S. military between August 1, 1990, and July 31, 1991, and enrolled in MVP were invited to participate in the survey via mail between June 2018 and March 2019. The overall survey response rate was 41.2% (44.4% for deployed and 40.3% for non-deployed Veterans) [25]. After excluding non-responders and individuals with incomplete responses, and considering MVP participants with available genotyping data [26] and genetically inferred ancestry classification [27], a total of 34,179 unrelated Veterans (European (EUR) descent *N* = 24,222; African (AFR) descent *N* = 6544; Admixed American (AMR) descent N = 2748; and East Asian (EAS) descent *N* = 665) were retained for the present study.

**Fig. 1:**
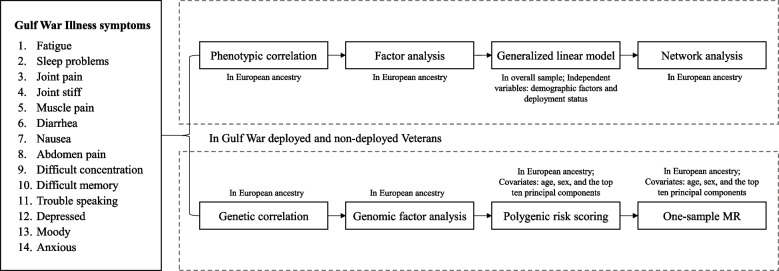
Flowchart of analyses conducted in the present study

All analyses were conducted separately in deployed (*N* = 11 736) and non-deployed Veterans (*N* = 22 443). We first described the distribution of 14 GWI symptoms (both presence and severity) and estimated their associations with demographic factors, deployment status, and GW-related exposures. In Veterans of genetically inferred EUR descent (the largest ancestry group available), we then performed correlation, factor, and network analyses to explore the underlying structure of the 14 symptoms. Lastly, we conducted polygenic risk score (PRS) and one-sample Mendelian randomization (MR) analyses to evaluate the potential causal effects of health-related traits on the symptom factors (Fig. 1).

### Gulf War Illness symptoms and independent variables

The GWI symptoms analyzed in this study were primarily based on the CDC case criteria [3], because of the previous evidence regarding its informativeness for GWI genetic studies [19, 28] and its ability to discriminate between deployed vs. non-deployed Veterans [20, 29]. CDC definition includes questions across three domains: fatigue; musculoskeletal symptoms (joint pain, joint stiffness, and muscle pain); and mood/cognition (sleep problems, difficulty with concentration, difficulty with memory, trouble finding words when speaking (hereafter referred to as trouble speaking), depressed, moody, and anxious) (Table S1) [3]. For diagnosis, difficulty with concentration and difficulty with memory will be combined into a single symptom category for defining GWI. However, we retained the questions as separate items in this study. Specifically, we used the CDC-GWI severe definition, which requires Veterans to report at least one symptom as ‘severe’ in two or more symptom domains. In addition to CDC-GWI symptoms, we also included three gastrointestinal symptoms (diarrhea, nausea, and abdominal pain) that are part of Kansas case definition, because they are highly associated with deployment status [4]. Thus, a total of 14 symptoms were assessed in our analysis (Table S1). For each symptom, study participants were asked: “Indicate No or Yes for each. Have you had a persistent or recurring problem with…?” If they responded “Yes”, they were further asked to rate the severity of the symptom (i.e., mild, moderate, or severe) and provide the year of onset.

Independent variables in this study included GW deployment status (deployed vs. non-deployed), as well as demographic and military characteristics, including sex, age, educational attainment, military rank, service branch, and Operation Enduring Freedom/Operation Iraqi Freedom (OEF/OIF) deployment (Table S1). For GW deployed Veterans, we also analyzed location within combat theater during GW deployment and eight self-reported GW-related exposures, including exposure to oil smoke, ground combat, use of PB anti-nerve agent pills, exposure to chemical or biological warfare (CBW) agents, work with prisoners of war, use of skin pesticides, wearing uniform treated with pesticides, and use of insect baits/no-pest strips. Individual-level data on the eight health-related phenotypes (anxiety, asthma, chronic pain, C-reactive protein (CRP), depression, neuroticism, PTSD, and type 2 diabetes (T2D)) investigated in the one-sample MR were extracted from self-reported information and VA electronic health records (EHR) from MVP. These phenotypes were selected because they showed significant associations with GWI in our previous GWAS [19].

### Genetic data

We leveraged individual-level genome-wide data from the MVP cohort. Details of genotyping, quality control (QC), and genetic imputation procedures have been described elsewhere [26]. Briefly, MVP participants were genotyped using a custom Axiom array and imputed using the Trans-Omics for Precision Medicine (TOPMed) reference panel [30]. In line with the National Academies of Sciences, Engineering, and Medicine for genetic and genomic research [31], we used genetically inferred ancestry groups as population descriptors. Ancestry groups (i.e., EUR, AFR, AMR, and EAS) were determined via a random forest clustering algorithm based on 1000 Genomes Project Phase 3 and Human Genome Diversity Project reference panels [27]. Here, AMR refers to the populations formed by the recent admixture of Native Americans, Europeans, and Africans following the colonization of the Americas [32]. PRS and one-sample MR analyses were performed only in EUR participants due to their largest sample size, applying QC criteria of removing samples with a missing rate > 10%, and genetic variants with imputation quality R^2^ < 0.8, minor allele frequency (MAF) < 1%, call rate < 90%, and Hardy–Weinberg equilibrium *P* < 1 × 10^–20^.

For genetically informed analysis, we used genome-wide association statistics derived from previously published studies in EUR-ancestry cohorts for the following eight phenotypes: anxiety, asthma [33], CRP [34], chronic pain [35], depression [36], neuroticism [37], PTSD [38], and T2D [39]. For anxiety, previously published GWAS summary statistics from Pan-UK Biobank (Phecode: 300) [40] and FinnGen (endpoint: KRA_PSY_ANXIETY) [41] were meta-analyzed. These external genome-wide association statistics were used only to derive PRS weights, and no individual-level data from these studies were used in the present study.

### Statistical analysis

To describe the distribution of GWI symptom presence, we reported the number and percentage of Veterans who reported each symptom. For symptom severity, we calculated the mean severity (coded as 0 = no symptom, 1 = mild, 2 = moderate, 3 = severe) and the percentage of Veterans at each severity level. For the year and age of symptom onset, we used quartiles (median, Q1, and Q3) to describe the distribution, and the Mann–Whitney U test was applied to assess differences by deployment status.

We evaluated phenotypic correlations among symptom pairs using the Phi coefficient for symptom presence and Kendall’s tau statistic for symptom severity. We also calculated single-nucleotide polymorphism (SNP)-based heritability and genetic correlation of symptoms using univariate and bivariate genomic restricted maximum likelihood (GREML) analyses [42, 43]. To estimate direct relationships among GWI symptoms, we performed network analysis using mixed graphical models (MGM), with extended Bayesian information criterion (BIC) used for model selection [44]. MGM is a probabilistic graphical model that captures conditional dependencies among variables of mixed data types (e.g., continuous, categorical, count data). MGM enables us to estimate and visualize edge weights in a symptom network, representing the strength of conditional correlations among symptoms. Given the sensitivity of network models to sample size, we applied a down-sampling strategy to the non-deployed group to match the sample size of the deployed group. We calculated three centrality indices for each symptom in the network: closeness (average closeness of a symptom to all other symptoms), betweenness (times a symptom lies on the shortest path between two other symptoms), and strength (sums of all edge weights of a symptom).

We used binary logistic regression models for symptom presence and ordinal logistic regression models for symptom severity to examine their associations with deployment status as well as demographic and military factors, including sex, age, ancestry, educational attainment, military rank, GW deployment location, and OEF/OIF deployment. For symptom presence, analyses were further stratified by deployment status (deployed vs. non-deployed) and symptom onset period (1990–2000 vs. post-2000). For the severity of each individual symptom, regression analyses were conducted in the full sample and repeated in Veterans who reported the symptom only. Severity analyses were also stratified by deployment status.

To assess potential non-linear associations, restricted cubic spline functions were incorporated into the regression models for symptom presence. Two models were considered: Model 1 adjusted for sex, ancestry, and deployment status (only in combined sample); Model 2 further adjusted for educational attainment, military rank, service branch, and eight self-reported GW-related exposures (only in deployed Veterans).

In addition, we applied latent class analysis (LCA) to classify all Veterans into 1–7 latent symptom classes based on the presence of 14 symptoms, selecting the optimal number of classes according to the minimum BIC. We also created a cumulative symptom score for every Veteran by summing the presence of 14 symptoms. Multinomial logistic regression for latent symptom classes and negative binomial regression for cumulative symptom score were then performed to estimate their associations with deployment status and demographic factors in all Veterans, and with eight GW-related exposures in deployed Veterans. Among deployed Veterans, analyses were further stratified by age group for cumulative symptom score. Variance inflation factors (VIF) were estimated to assess multicollinearity in multivariable analyses.

We employed a three-step strategy in the factor analyses to assess the latent structure underlying GWI symptoms. First, we performed Bartlett’s test and computed an overall measure of sampling adequacy (MSA) in Kaiser–Meyer–Olkin (KMO) test to assess the suitability of the symptom data for factor analysis [45]. For data deemed appropriate, we randomly split the dataset into two halves. In the first half, we conducted parallel analysis and exploratory factor analysis (EFA) to determine the optimal number of latent factors and their structure. In the second half, confirmatory factor analysis (CFA) was subsequently performed to estimate factor loadings. To quantify genetic and environmental influences on latent factors, we implemented genomic CFA using the genomic-relatedness-matrix restricted maximum-likelihood method via the OpenMx R package (mxGREML). This approach estimates the additive-genetic variance (Vac) and nonshared-environmental variance (1 − Vac), the latter reflecting individual-specific environmental exposures, even within families [46]. In this study, environmental exposures included both the eight GW-related exposures described above, as well as other GW-related or non-GW-related exposures. To address the substantial computational burden of mxGREML, the dataset was randomly partitioned into non-overlapping subgroups of approximately 500 Veterans each. We fitted mxGREML within each subgroup, and the results were pooled using a random-effects meta-analysis [47].

To assess the putative causal relationships between symptom factors derived from CFA and the eight health-related outcomes previously associated with CDC-GWI [19], we performed PRS and one-sample MR analyses in deployed and non-deployed Veterans, with sex, age, and the top ten within-ancestry principal components (PC) as covariates. We also examined the associations between latent symptom classes and PRSs of the eight health-related outcomes in deployed Veterans. PRS analysis quantifies the aggregate genetic liability to each health-related phenotype and tests whether this liability is associated with GWI symptom factors or classes. PRS weights were estimated using the previously calculated genome-wide association statistics (see Genetic Data) via Polygenic Risk Score–Continuous Shrinkage (PRS-CS) method [48], using the UK Biobank EUR-ancestry participants as the linkage disequilibrium (LD) reference panel. Individual PRSs were computed using PLINK 2.0 [49]. To enable comparison of effect sizes across different analyses, both PRSs and GWI symptom factor scores were standardized (z-score: mean = 0, SD = 1), and their associations were evaluated via logistic regression. One-sample MR extends PRS analysis by using genetic liability as an instrumental variable to estimate putative causal effects of health-related outcomes on symptom factors, employing the two-stage least-squares (2SLS) method implemented in *ivreg* R package [50]. All variables in the model were standardized (z-score: mean = 0, SD = 1) prior to regression to facilitate effect size comparison. Weak-instrument and Wu-Hausman tests were used to evaluate instrument strength and model validity.

Unless otherwise specified, Bonferroni corrections were applied within each analysis to account for multiple testing. All analyses and result visualizations were performed in R (version 4.3.2).

## Results

### Symptom distribution and correlation

**Fig. 2:**
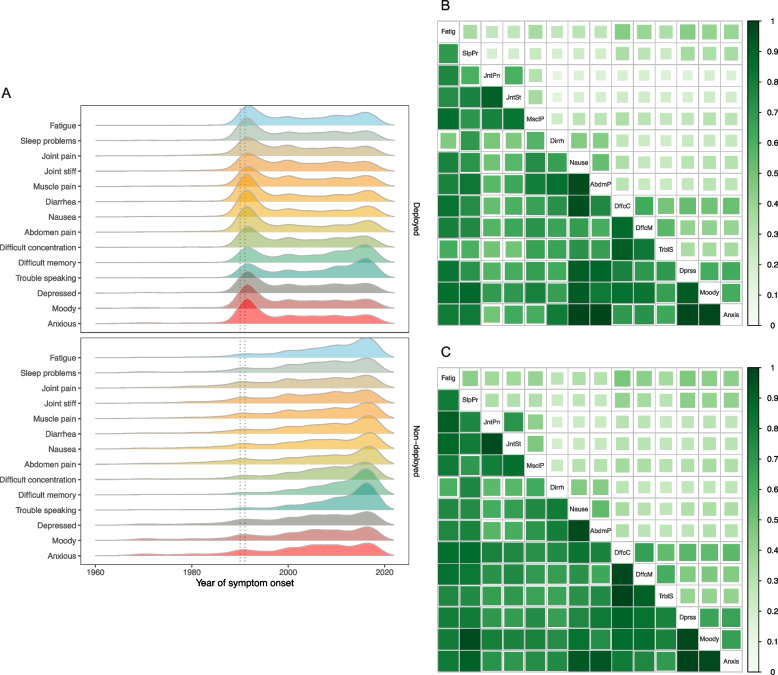
Year of onset and correlation of Gulf War Illness Symptoms. **A** Distribution of symptom onset year in Gulf War deployed and non-deployed Gulf War Era Veterans. The colored areas represent the density distributions of each symptom estimated using a Gaussian kernel density estimator with the bandwidth set to 0.9 times the minimum of the standard deviation and the interquartile range divided by 1.34 times the sample size to the negative one-fifth power. The two vertical dashed lines mark years 1990 and 1991. **B** Phenotypic (upper right) and genetic (lower left) correlations of symptom presence in the overall sample. Phenotypic correlation was measured by Phi coefficient. **C** Phenotypic (upper right) and genetic (lower left) correlations of symptom severity in the overall sample. Phenotypic correlation was measured by Kendall’s tau. In B and C, both the color and size of the squares represent the magnitude of the correlation coefficient. Symptom abbreviations: Fatig, fatigue; SlpPr, sleep problems; JntPn, joint pain; JntSt, joint stiffness; MsclP, muscle pain; Dirrh, diarrhea; Nause, nausea; AbdmP, abdomen pain; DffcC, difficulty with concentration; DffcM, difficulty with memory; TrblS, trouble finding words when speaking; Dprss, depressed; Anxis, anxious

This study included a total of 34,179 Veterans (16% female, 34% deployed, mean age 61 ± 8 years; Table S2). Among the 14 GWI symptoms, joint pain had the highest prevalence at 82% (86% in deployed Veterans and 79% in non-deployed Veterans), while the three gastrointestinal symptoms had the lowest prevalence (all < 25%; Table S2; Fig. S1). Regarding severity, a similar pattern was observed, with joint pain showing the highest mean severity (1.67) and the largest proportion of Veterans rated as “severe” (23.7%) (Table S3; Fig. S2). However, looking only at Veterans with the symptom, sleep problems showed the greatest severity in both sexes, deployed Veterans, and the three youngest age groups (Table S3). When comparing the distribution of the onset year across the 14 symptoms, we found significant differences between deployed and non-deployed Veterans (Fig. 2A; Table S4). As shown in Fig. 2A, deployed Veterans exhibited the greatest and most consistent symptom onset across all 14 symptoms in the early 1990s, followed by a more gradual increase over time after 2000. This early-1990s peak was observed in both CDC-GWI severe cases and controls among deployed Veterans, and was particularly evident in younger age groups (Fig. S3 and S4). In contrast, symptom onset was relatively infrequent before 2000 among non-deployed Veterans and increased after 2000. Deployed Veterans had statistically earlier onset years for all 14 symptoms than non-deployed (*P* < 0.004). Similar differences were found for the distribution of age of symptom onset (*P* < 0.004; Table S4; Fig. S5).

Bonferroni-significant phenotypic correlations (*P* < 5.5 × 10^–4^) were present among GWI symptoms, considering both presence and severity correlation coefficients larger in deployed Veterans than non-deployed Veterans (Fig. 2B and C; Table S5; Fig. S6–S9). The 14 GWI symptoms phenotypically clustered into three domains: gastrointestinal, musculoskeletal, and other symptoms (e.g., fatigue, cognitive, and mood symptom categories) (Fig. S6–S9). Genetic correlations for both symptom presence and severity were also statistically significant in the whole sample (Fig. 2B and C; Table S5). However, the sample size available limited the statistical power of the deployment-stratified analyses (Table S5; Fig. S10–S13). Similarly, the SNP-based heritability estimates of the 14 symptoms were significant in the whole sample (*P* < 0.004; Table S6), while the deployment-stratified analysis showed statistical significance only for certain symptoms (e.g., fatigue presence in deployed Veterans: h^2^ = 0.152, P = 1.67 × 10^–4^; fatigue presence in non-deployed Veterans: h^2^ = 0.132, P = 5.17 × 10^–10^), with a greater number of significant estimates in non-deployed than deployed Veterans.

### Symptom-level associations

**Fig. 3:**
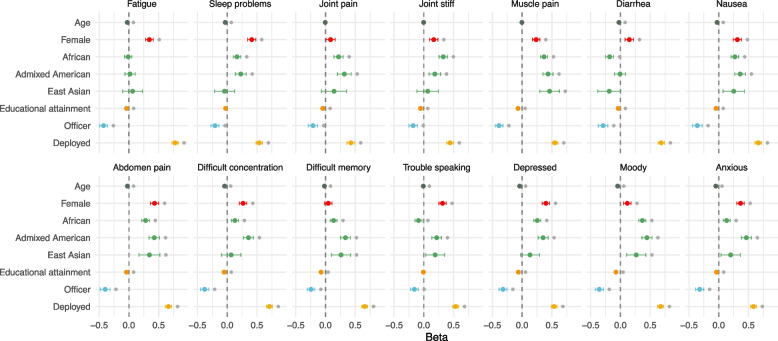
Associations of Gulf War Illness symptom presence with demographics and deployment status. Multivariable logistic regression models were performed to assess the associations of symptom presence with demographic factors and deployment status. The reference group for sex, ancestry, military rank, and deployment status were male, European ancestry, enlisted Veterans, and non-deployed Gulf War Era Veterans, respectively. Grey star symbols indicate associations surviving Bonferroni multiple testing correction

In the multivariable logistic regression analyses, we identified statistically significant associations of demographic factors and GW deployment status with the presence of all 14 symptoms (Fig. 3; Table S7). Specifically, female sex, non-EUR ancestry, and GW deployment were associated with higher symptom frequencies, while older age, higher educational attainment, and officer rank were associated with lower symptom frequencies (*P* < 0.004). An exception was observed for Veterans of AFR-descent, who had a lower prevalence of diarrhea and trouble speaking compared to Veterans of EUR-descent. Additionally, adjusting for OEF/OIF deployment did not change the pattern of the associations described above (Table S8). In analyses stratified by deployment status, the associations of AFR ancestry with sleep problems and several cognitive and mood symptoms were stronger in deployed Veterans than non-deployed Veterans (*P* < 0.004; Table S9; Fig. S14). In analyses stratified by year of onset, the associations between GW deployment and several demographics (e.g., age, female, AFR ancestry, educational attainment, and officer rank) were stronger for symptoms with onset during 1990–2000 than for those with onset after 2000 (*P* < 0.004; Table S10; Fig. S15). We further stratified the analyses by both year of onset and GW deployment status. In analyses for symptom onset year during 1990–2000 in deployed Veterans, deployment to Iraq or Kuwait (vs. at sea or on ship) was associated with increased presence of all 14 symptoms (*P* < 0.004; Table S11; Fig. S16). However, the negative relationship of AFR ancestry with diarrhea mentioned above was only significant for symptoms with onset during 1990–2000, while the negative association with trouble speaking was only significant after 2000 in non-deployed Veterans (beta = −0.160, *P* = 7.8 × 10^–5^; Table S11; Fig. S16).

Younger age was associated with a higher prevalence of GWI symptoms after adjustment for covariates. To investigate further this inverse relationship, we modeled potential nonlinear associations while also considering additional covariates. In the combined sample and in non-deployed group, most nonlinear terms indicated that older age was associated with decreased symptom presence (*P* < 0.05), and the effect sizes were slightly attenuated after adjusting for educational attainment, military rank, and service branch (Table S12; Fig. S17 and S18). In deployed Veterans, the inverse relationship was further attenuated after adjusting also for eight self-reported GW-related exposures, with the association of muscle pain even changing its direction to positive—older age was now associated with a higher prevalence of muscle pain (Fig. S19).

Regarding symptom severity, multivariable regression revealed associations similar to those for symptom presence (Table S13). For individual symptoms, when excluding Veterans without each symptom, the effect sizes were attenuated compared to analyses including all Veterans (P < 0.004; Table S13; Fig. S20). However, exceptions were observed for AFR ancestry, where several effect sizes increased after the exclusion, whereas many associations of EAS ancestry shifted to negative. Comparisons (including Veterans both with and without each symptom) between deployed and non-deployed Veterans also showed patterns consistent with those from the symptom presence analysis (Table S14; Fig. S21).

LCA identified seven latent symptom classes among all Veterans, based on the minimum BIC. Class 1 was characterized by high probabilities across all 14 symptoms; Class 2 by high probabilities for all except gastrointestinal symptoms; Class 3 by fatigue/sleep, cognitive, and mood symptoms; Class 4 by fatigue/sleep, musculoskeletal, and cognitive symptoms; Class 5 by fatigue/sleep, musculoskeletal, and mood symptoms; Class 6 by musculoskeletal symptoms only; and Class 7 by uniformly low probabilities across all symptoms (Fig. S22). Multivariable multinomial logistic regression in all Veterans confirmed most demographic associations in classes 1 and 2, while officer rank and GW deployment were significant across classes 1 through 5 (Table S15). When investigating eight GW-related exposures in deployed Veterans, univariable analyses found most exposures significantly associated with all classes, with exceptions in classes 3 and 6, while distinct exposure patterns were observed across classes in multivariable analyses. For instance, exposure to CBW agents was associated with classes 1, 2, 4, and 5, whereas exposure to PB anti-nerve agent pills was exclusively associated with class 3. Regarding cumulative symptom score, multivariable negative binomial regression in all Veterans identified significant associations for all demographics and GW deployment; however, ancestry was no longer significant in the 40–50 age group or after adding eight GW-related exposures in deployed Veterans (Table S16). Age-group stratified analyses among deployed Veterans showed that exposure to oil smoke was significant only in the younger 40–55 age groups, whereas exposures to CBW agents (40–75) and pesticide cream (40–55 and 65–75) were significant in most age groups. For all multivariable analyses including those related to GW-related exposures, VIF values were below 2.5, indicating low multicollinearity.

### Structure analysis of GWI symptoms

**Fig. 4:**
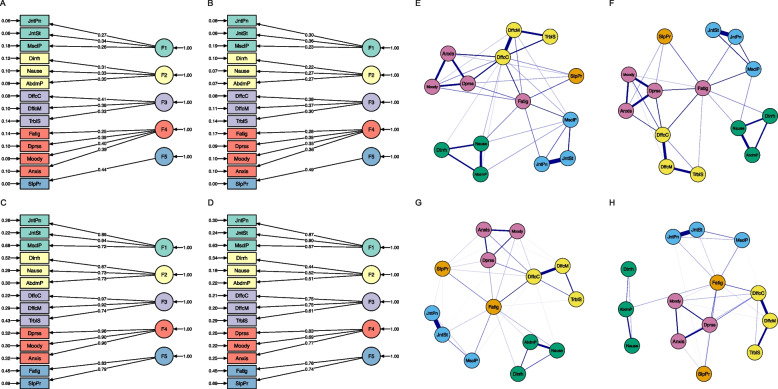
Factor and network analyses of Gulf War Illness symptoms. Confirmatory factor analysis (CFA) was performed for symptom presence in Gulf War deployed (**A**) and non-deployed Gulf War Era (**B**) Veterans, and for symptom severity in deployed (**C**) and non-deployed (**D**) Veterans. In panels A–D, circles represent latent factors (F1–F5), and rectangles represent 14 observed symptom variables. Numbers on arrows from circles to rectangles indicate factor loadings, while numbers attached to rectangles indicate residual variances. Numbers attached to circles indicate that factor variances were fixed to 1. Network analysis was conducted for symptom presence in deployed (**E**) and non-deployed (**F**) Veterans, and for symptom severity in deployed (**G**) and non-deployed (**H**) Veterans. Edges in the networks represent direct correlations between symptoms. Node colors indicate factors identified by the CFA. Abbreviations: Fatig, fatigue; SlpPr, sleep problems; JntPn, joint pain; JntSt, joint stiffness; MsclP, muscle pain; Dirrh, diarrhea; Nause, nausea; AbdmP, abdomen pain; DffcC, difficulty with concentration; DffcM, difficulty with memory; TrblS, trouble finding words when speaking; Dprss, depressed; Anxis, anxious; F1, musculoskeletal factor; F2, gastrointestinal factor; F3, cognitive factor; F4, mood factor; F5, fatigue/sleep factor

Bartlett’s test (*P* < 2.2 × 10^–16^) and overall KMO MSA (> 0.88) indicated that GWI symptoms data were suitable for factor analysis [45]. Accordingly, we conducted phenotypic EFA to evaluate one- to five-factor models of the 14 symptoms. We then built phenotypic CFA models based on these structures (Table S17). Across all five models, both the parallel analysis (Fig. S23–S26) and CFA goodness-of-fit indices (Table S18) indicated that the five-factor model provided the best fit for symptom presence. The five factors were interpreted as musculoskeletal, gastrointestinal, cognitive, mood, and sleep factors according to EFA results (Fig. 4A–D). For symptom severity, the fifth factor also included fatigue rather than only sleep problems (i.e., fatigue/sleep factor). In all CFA models, symptoms showed significant factor loadings (*P* < 0.01), with generally higher loadings in deployed group compared to non-deployed group (e.g., Z of 11 significant differences ranging from 3.1 to 11.8 in the five-factor model of symptom severity; Table S17). Genomic CFA suggested lower additive-genetic variance for most factors in deployed group compared to non-deployed group. However, most of these genetic components and deployment-related differences did not reach statistical significance, likely due to the small sample size and inherent limitations of the genomic CFA approach (Table S19).

Network analysis showed that fatigue had the highest closeness and betweenness indices, emerging as the central node in networks of both deployed and non-deployed Veterans (Fig. 4E–H; Table S20; Fig. S27–S30). The symptom network was denser (i.e., having higher number of edges) among deployed group, with 34 (mean weight = 0.14) and 37 (mean weight = 0.073) non-zero edges for symptom presence and severity, respectively, compared to 28 (mean weight = 0.12) and 30 (mean weight = 0.070) in non-deployed group. For instance, non-deployed network for symptom presence lacked direct correlations of difficulty with memory with fatigue (weight: 0.208 in deployed vs. 0 in non-deployed) and muscle pain (0.095 vs. 0), as well as difficulty with concentration with sleep problems (0.185 vs. 0) and nausea (0.123 vs. 0) (Fig. 4E–H; Table S20).

### Pleiotropy analysis

**Fig. 5:**
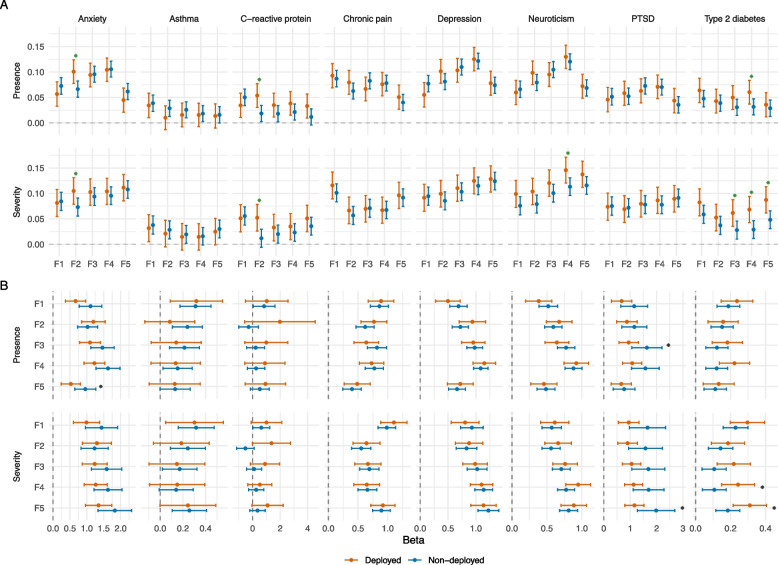
Polygenic risk score (**A**) and one-sample Mendelian randomization (**B**) analyses for symptom factors of Gulf War Illness and other complex traits. All models were adjusted for sex, age, and the top ten principal components. Green star markers indicate nominally significant (*P* < 0.05) differences in effect sizes between Gulf War deployed and non-deployed Gulf War Era Veterans. F1, musculoskeletal factor; F2, gastrointestinal factor; F3, cognitive factor; F4, mood factor; F5: fatigue/sleep factor

All PRS and one-sample MR analyses were conducted in EUR-descent Veterans only, because the sample sizes in the other ancestry groups were insufficient for these analyses. The PRSs of the eight health-related phenotypes tested showed statistically significant associations (*P* < 0.001) with the five symptom factors, except for certain models related to asthma and CRP PRSs (Fig. 5A; Table S21). Regarding deployment status, the effect sizes were nominally larger in deployed Veterans than in non-deployed Veterans in nine models (*P* < 0.05). These included associations of anxiety and CRP PRSs with the gastrointestinal factor, T2D PRS with the cognitive, mood, and sleep factors, and neuroticism PRS with the mood factor (Fig. 5A; Table S21). Regarding latent symptom classes in deployed Veterans, most PRSs were significantly associated with classes 1 and 2, with the exceptions of asthma and CRP PRSs (Table S22). Class 5 was associated with anxiety, chronic pain, depression, and neuroticism PRSs, while other classes showed more selective patterns: class 3 was associated only with depression PRS, and classes 4 and 6 only with chronic pain PRS.

The one-sample MR analysis showed putative causal effects of the health-related phenotypes on GWI-symptom factors, again with the exception of models related to asthma and CRP (Fig. 5B; Table S23). Five models showed nominally significant differences in causal effect sizes between deployed and non-deployed Veterans (*P* < 0.05; Fig. 5B; Table S23). Specifically, the causal effects of T2D on mood and sleep factors were larger in the deployed group. In contrast, the causal effects of PTSD on cognitive and sleep factors, and of anxiety on the sleep factor, were larger in the non-deployed group. The sensitivity analyses supported the reliability of most instrumental variable estimations based on weak-instrument and Wu-Hausman tests (*P* < 0.05), except for models primarily involving asthma and CRP showing limited strength for instruments.

## Discussion

The present study advances GWI symptom-level research by comprehensively characterizing the prevalence, severity, onset patterns, and internal structure of GWI symptom profiles in relation to demographic and military characteristics, as well as health-related phenotypes. Among the 14 GWI symptoms investigated, joint pain and joint stiffness exhibited the highest prevalence in line with the frequently reported musculoskeletal issues among GW-Era Veterans [51] and in older populations [52]. Regarding symptom severity, sleep problems were the most severe symptom, suggesting that although musculoskeletal symptoms are highly prevalent, sleep problems may have a greater impact on Veterans’ health and life quality [53].

The bimodal year- and age-of-onset distributions among GWI symptoms observed in the present study identified consistent differences between deployed and non-deployed Veterans. Specifically, all symptoms first occurred at significantly younger ages and in closer proximity to the Persian Gulf War (1990–1991) among deployed Veterans compared to non-deployed Veterans. This is consistent with previous indications that GWI developed in response to Gulf War deployment in 1990–1991 [2, 9, 20, 54, 55]. In contrast, symptom onset in non-deployed Veterans increased more gradually over time, consistent with a pattern that may reflect age-related symptom accrual.

Our findings highlight the underlying symptom structure, the central role of fatigue in symptom connections, and the uneven contribution of environmental factors between deployment groups. Specifically, GWI symptoms showed a high correlation in both phenotypic and genetic analyses, suggesting shared mechanisms underlying GWI pathogenesis. However, the sample size available did not permit us to fully investigate the genetic overlap among GWI symptoms in deployed vs. non-deployed Veterans. Phenotypic EFA identified five latent factors (i.e., musculoskeletal, gastrointestinal, cognitive, mood, and fatigue/sleep factors) underlying the 14 GWI symptoms investigated. Notably, when modeling symptom severity, sleep problems and fatigue loaded onto a combined factor, whereas in the symptom-presence EFA, sleep problems loaded separately. This difference can be attributed to the fact that the ordinal severity data contains more information than binary presence data and thus yields higher statistical power [56]. In the CFA analysis, significant differences were observed between deployed and non-deployed Veterans, with most factor loadings being higher in deployed group. This suggests stronger associations among GWI symptoms and their latent factors in deployed Veterans, despite the smaller sample size of this group [57]. In the genomic CFA, the genetic contributions to most factors were lower in the deployed group, implying a greater influence of environmental exposures [54]. This pattern is also consistent with the lower number of symptoms with statistically significant SNP-based heritability observed in deployed Veterans. Network analysis revealed denser direct connections among the symptoms in deployed than non-deployed group, reflecting greater interdependence among symptoms and higher potential vulnerability in deployed group, where dysfunction in a few key biological mechanisms may trigger widespread symptom manifestation and contribute to the development of GWI [58, 59]. Among the 14 symptoms, fatigue has the largest closeness and betweenness indices in the network, consistent with previous reports and reinforcing its recognized clinical importance [59–61].

The present study also highlights the long-term impact of deployment on GWI symptoms. Specifically, GW deployment was associated with both early symptom onset (occurred between years 1990 and 2000) and late symptom onset (after year 2000), strengthening prior evidence on the GWI-GW deployment relationship [20, 54]. Demographic factors were also significant for post-2000 symptom onset in both deployed and non-deployed Veterans. For instance, stronger associations between female sex and GWI symptoms may reflect physiological sensitivity to GW-related exposures and sex hormone differences [20, 62]. Similarly, the protective effect of educational attainment with respect to most GWI symptoms may be related to the lower environmental and duty-related exposure levels experienced and the greater access to support resources in officer rank vs. enlisted Veterans [54]. In general, non-EUR groups had higher symptom prevalence, which may reflect differences in environmental and duty-related exposures, genetic susceptibility, and healthcare access [63]. One exception was the relationship between AFR ancestry and a lower prevalence of diarrhea reported with onset before year 2000. While genetic predisposition may underlie this protective association, it could also be due to differences in exposures, behavioral factors, and gastrointestinal resilience [64, 65]. AFR ancestry was also associated with a lower prevalence of trouble speaking with onset after year 2000 in non-deployed Veterans, which may reflect differences in underlying risk factor profiles between deployed and non-deployed groups [4]. Additionally, sociocultural differences in symptom perception and reporting across ancestry groups may also contribute to these negative associations between AFR ancestry and symptoms [66, 67]. We also observed that among deployed Veterans, those located in Iraq or Kuwait reported more symptoms than those located primarily at sea/on ship, with the former being locations where nearly all battles had taken place and where the highest rates of Veteran-reported GW-related exposures occurred [54]. An inverse relationship was observed between older age and lower prevalence of most GWI symptoms, which our non-linear association analysis suggested were driven by socioeconomic factors, military rank, and GW-related exposures that likely differed by age during the time of GW [20]. In addition, GW-related exposures showed broad but heterogeneous associations with latent symptom classes and cumulative symptom score. Although the underlying mechanisms remain unclear, these exposures may contribute to symptom burden through neurobiological effects involving subcortical brain systems, which is broadly consistent with the brain-region enrichment signals observed in our previous GWI GWAS study [19].

PRS and one-sample MR analyses highlighted the direct effect of the genetic liability to health-related phenotypes on GWI-symptom factors. However, we observed that asthma and CRP may be less strongly associated with certain GWI symptom domains than we observed previously with respect to CDC-GWI case–control definitions [19]. Previous studies highlighted the complex interplay of asthma and CRP with comorbid conditions affecting Veterans [22, 68]. Interestingly, we observed that T2D effect on GWI-symptom factors was larger in deployed than in non-deployed, while PTSD effect on GWI-symptom factors was larger in non-deployed than in deployed. As previously hypothesized [19], this may indicate that genetic liability to GWI in deployed may be more related to metabolic health, while GWI symptoms in non-deployed may be linked to central nervous system effects. Our previous gene-by-environment study further reported that the association between T2D PRS and GWI in deployed Veterans remained significant after controlling for GW-related exposures and T2D diagnosis [28]. In addition, non-deployed Veterans may have experienced other psychological stress, such as sexual trauma, motor vehicle accidents, and anticipating potential combat deployment, which may have contributed to the interplay between PTSD PRS and GWI symptom burden, although this hypothesis requires further investigation.

Our study has several limitations. First, although the cohort investigated is the largest available to study the underlying structure and genetic liability of GWI to date, the sample size was still insufficient to conduct certain genetically informed analyses, such as statistically powerful GWAS of GWI symptoms and PRS or MR analyses in non-EUR ancestry groups. The lack of symptom-level GWAS data did not permit us to use genomic structural equation modeling to characterize the genetic architecture of GWI symptoms [69]. The sample size also limited the genetic correlation analyses and the regression analyses involving CRP. Unlike self-reported data, CRP measurements were available for only a small subset of participants (*N* = 7805). In addition to the limited understanding of GWI etiology and the lack of consensus on case definition, environmental exposures in combat theater were not systematically recorded and also relied on retrospective self-reporting. These methodological limitations may introduce information bias and misclassification, potentially distorting the associations [14]. Moreover, our analysis primarily focused on GWI symptoms defined by the CDC case definition; we did not include all symptoms from the Kansas case definition, such as respiratory symptoms. In addition, because the ICD-10-CM GWI diagnosis code was not available until October 2025, we could not investigate this definition in the present study. Finally, with respect to deployed vs. non-deployed comparisons, the imbalanced sample sizes available for these groups may also have partially contributed to the differences observed [70].

## Conclusions

This study demonstrated that deployed GW Veterans had earlier symptom onset, greater symptom burden, and stronger symptom interrelationships than non-deployed Veterans. We identified a five-factor latent structure underlying GWI symptoms and found fatigue to have the largest closeness and betweenness in the symptom network. Genetically informed analyses further indicated that GWI symptom factors are linked to pleiotropic liability involving both physical and mental health-related phenotypes, with different patterns observed in deployed and non-deployed Veterans. These findings advance the symptom-level characterization of GWI, help clarify its heterogeneity and potential pathobiology, and may inform a refined case definition and targeted symptom-based management strategies for Gulf War Veterans. Future studies should investigate the long-term trajectories of GWI and related health outcomes and identify potential therapeutic targets and drug candidates for GWI treatment.

## Supplementary Information

Supplementary Material 1 - Supplementary Figures.

Supplementary Material 2 - Supplementary Tables.

Supplementary Material 3 - MVP Core Acknowledgements.

## Data Availability

Data is provided within the manuscript or supplementary information files.

## Abbreviations

AFR: African
AMR: Admixed American
CBW: Chemical or biological warfare
CDC: Centers for Disease Control and Prevention
CFA: Confirmatory factor analysis
CRP: C-reactive protein
EAS: East Asian
EFA: Exploratory factor analysis
EHR: Electronic health records
EUR: European
GW: Gulf War
GWAS: Genome-wide association study
GWI: Gulf War Illness
ICD-10-CM: International Classification of Diseases, 10th Revision, Clinical Modification
LCA: Latent class analysis
LD: Linkage disequilibrium
MAF: Minor allele frequency
MR: Mendelian randomization
MVP: Million Veteran Program
OEF/OIF: Operation Enduring Freedom/Operation Iraqi Freedom
PB: Pyridostigmine bromide
PC: Principal component
PRS: Polygenic risk score
PTSD: Posttraumatic stress disorder
QC: Quality control
SNP: Single-nucleotide polymorphism
T2D: Type 2 diabetes
VA: Department of Veterans Affairs
VIF: Variance inflation factor
