# Supplementary material for: Observational and genetically-informed characterization of Gulf War Illness symptoms in 1990–1991 Gulf War deployed Veterans and non-deployed Gulf War Era Veterans
Source: Environ Health. 2026 Jun 11;25:71. doi: 10.1186/s12940-026-01316-5 (PMC13479420; doi:10.1186/s12940-026-01316-5)
Supplement: Supplementary file 1 [file 12940_2026_1316_MOESM1_ESM.pdf]

Supplementary Figures

Observational and genetically-informed characterization of Gulf War Illness symptoms in 1990–1991 Gulf War deployed Veterans and non-deployed Gulf War Era Veterans.

Figure S1. Prevalence of Gulf War Illness symptom presence
Figure S2. Distribution of Gulf War Illness symptom severity
Figure S3. Distribution of symptom onset year in Gulf War deployed and non-deployed Gulf War Era Veterans, stratified by case-control status
Figure S4. Distribution of symptom onset year in Gulf War deployed and non-deployed Gulf War Era Veterans, stratified by age group
Figure S5. Distribution of onset age for Gulf War Illness symptoms
Figure S6. Phenotypic correlations between pairs of Gulf War Illness symptom presence in Gulf War deployed Veterans
Figure S7. Phenotypic correlations between pairs of Gulf War Illness symptom presence in Gulf War non-deployed Veterans
Figure S8. Phenotypic correlations between pairs of Gulf War Illness symptom severity in Gulf War deployed Veterans
Figure S9. Phenotypic correlations between pairs of Gulf War Illness symptom severity in Gulf War non-deployed Veterans
Figure S10. Genetic correlations between pairs of Gulf War Illness symptom presence in Gulf War deployed Veterans
Figure S11. Genetic correlations between pairs of Gulf War Illness symptom presence in Gulf War non-deployed Veterans
Figure S12. Genetic correlations between pairs of Gulf War Illness symptom severity in Gulf War deployed Veterans
Figure S13. Genetic correlations between pairs of Gulf War Illness symptom severity in Gulf War non-deployed Veterans
Figure S14. Gulf War deployment specific associations of Gulf War Illness symptom presence with demographics
Figure S15. Associations of Gulf War Illness symptom presence with demographics and deployment status stratified by symptom onset year
Figure S16. Associations of Gulf War Illness symptom presence with demographics stratified by symptom onset year and deployment status
Figure S17. Non-linear associations between age and Gulf War Illness symptom presence in overall Veterans

Figure S18. Non-linear associations between age and Gulf War Illness symptom presence in Gulf War non-deployed Veterans
Figure S19. Non-linear associations between age and Gulf War Illness symptom presence in Gulf War deployed Veterans
Figure S20. Associations of Gulf War Illness symptom severity with demographics and Gulf War deployment status in all Veterans and Veterans with a specific symptom
Figure S21. Gulf War deployment specific associations of Gulf War Illness symptom severity with demographics
Figure S22. Latent symptom classes in all Veterans identified by latent class analysis based on the presence of 14 Gulf War Illness symptoms.
Figure S23. Parallel analysis scree plot for Gulf War Illness symptom presence in Gulf War deployed Veterans
Figure S24. Parallel analysis scree plot for Gulf War Illness symptom presence in Gulf War non-deployed Veterans
Figure S25. Parallel analysis scree plot for Gulf War Illness symptom severity in Gulf War deployed Veterans
Figure S26. Parallel analysis scree plot for Gulf War Illness symptom severity in Gulf War non-deployed Veterans
Figure S27. Centrality indices for the network of Gulf War Illness symptom presence in Gulf War deployed Veterans
Figure S28. Centrality indices for the network of Gulf War Illness symptom presence in Gulf War non-deployed Veterans
Figure S29. Centrality indices for the network of Gulf War Illness symptom severity in Gulf War deployed Veterans
Figure S30. Centrality indices for the network of Gulf War Illness symptom severity in Gulf War non-deployed Veterans

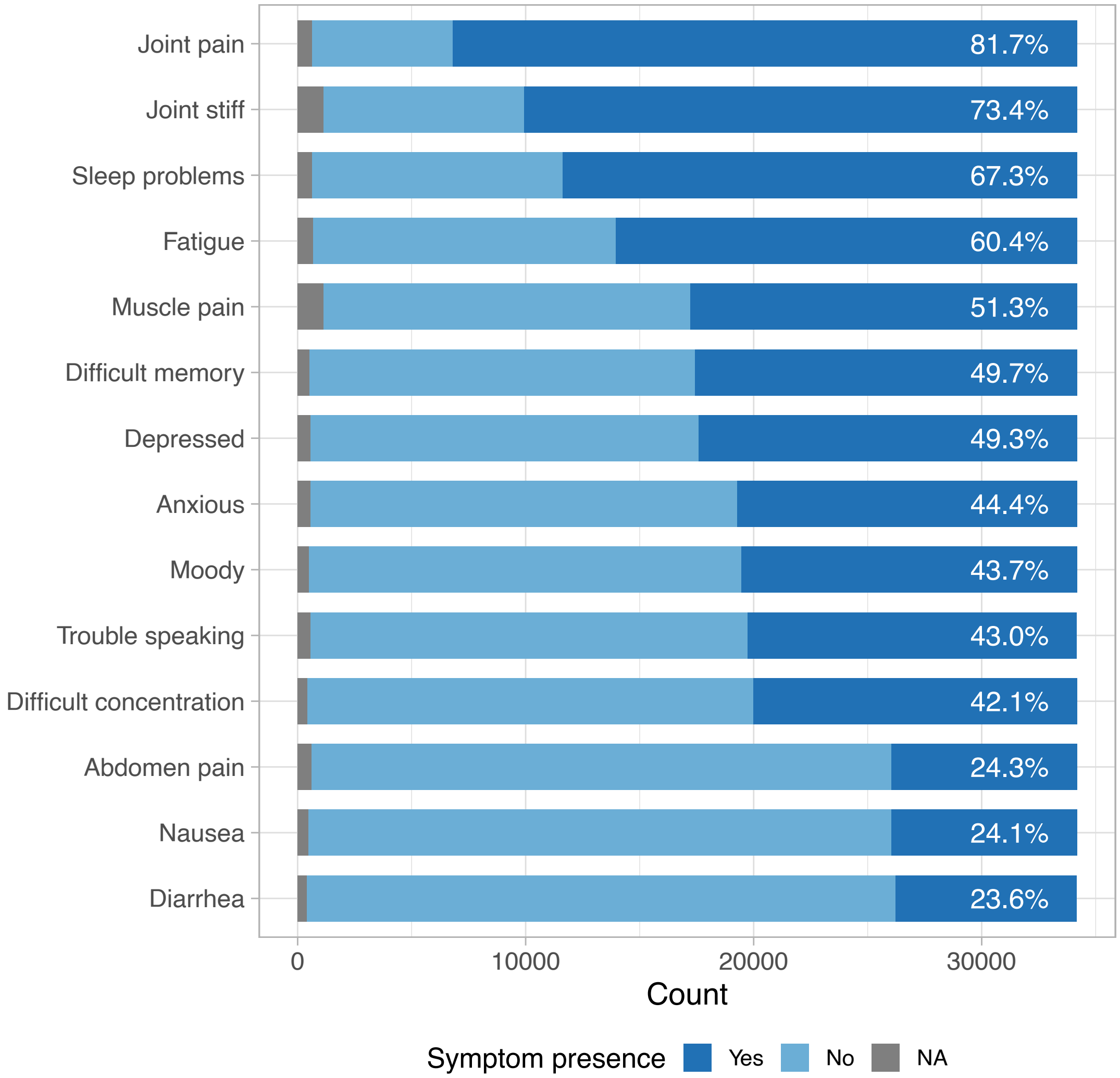

S1. Prevalence of Gulf War Illness symptom presence. Trouble speaking, trouble finding words when speaking.

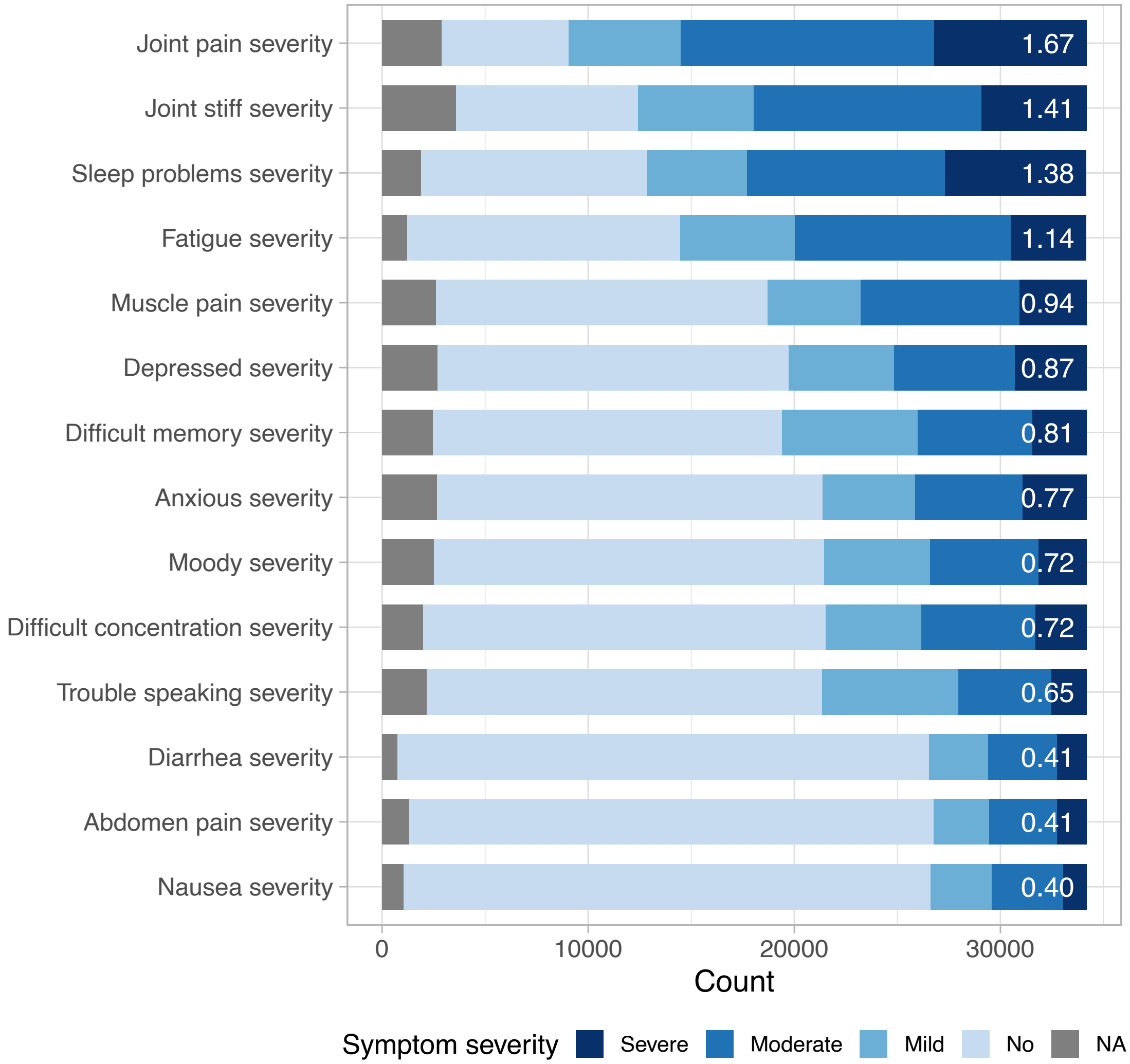

S2. Distribution of Gulf War Illness symptom severity. The numbers within each bar represent the mean symptom severity, coded as follows: 0 = 'No', 1 = 'Mild', 2 = 'Moderate', and 3 = 'Severe'. Trouble speaking, trouble finding words when speaking.

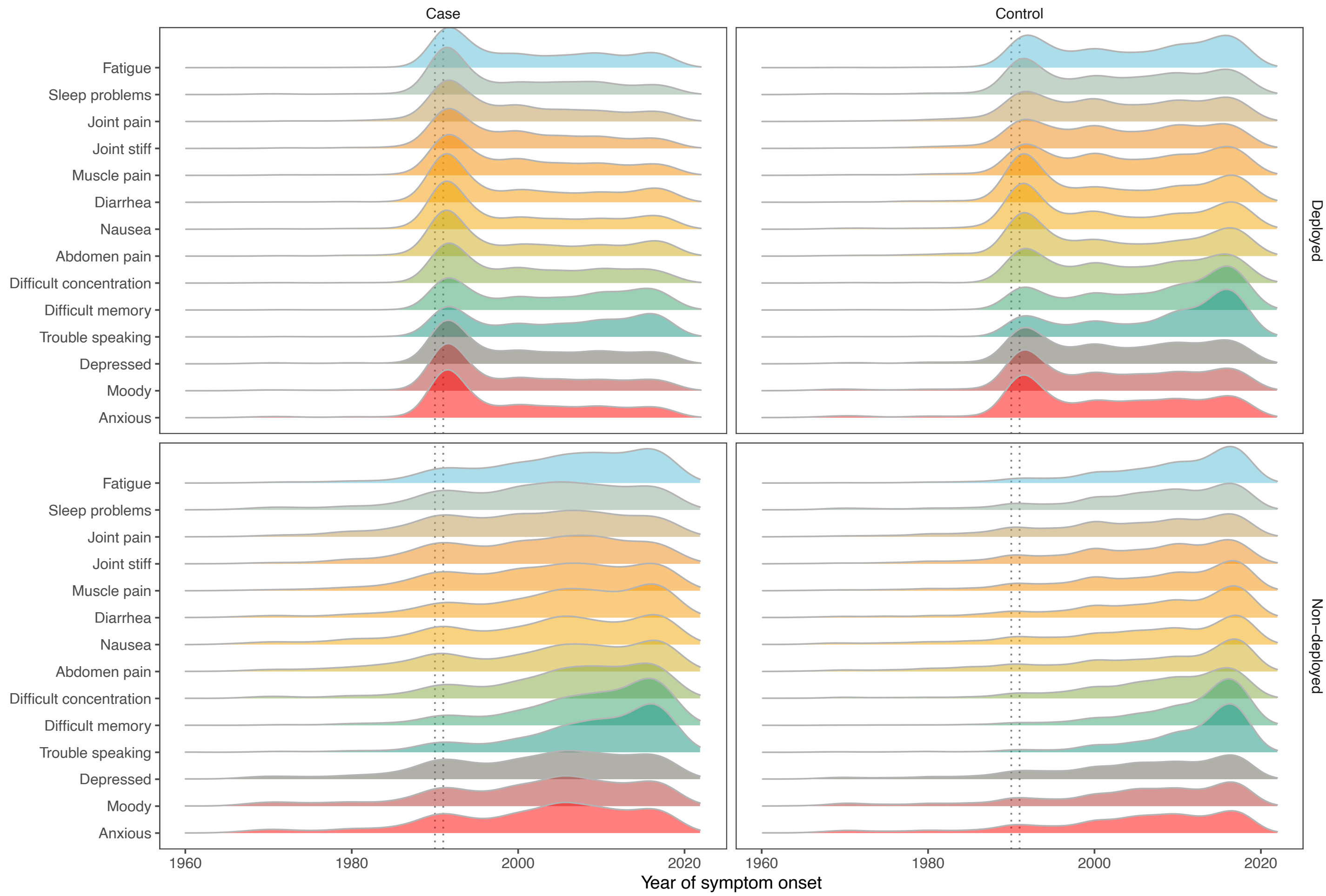

S3. Distribution of symptom onset year in Gulf War deployed and non-deployed Gulf War Era Veterans, stratified by CDC-Gulf War Illness severe case-control status. The colored areas represent the density distributions of each symptom estimated using a Gaussian kernel density estimator with the bandwidth set to 0.9 times the minimum of the standard deviation and the interquartile range divided by 1.34 times the sample size to the negative one-fifth power. The two vertical dashed lines mark years 1990 and 1991. Trouble speaking, trouble finding words when speaking.

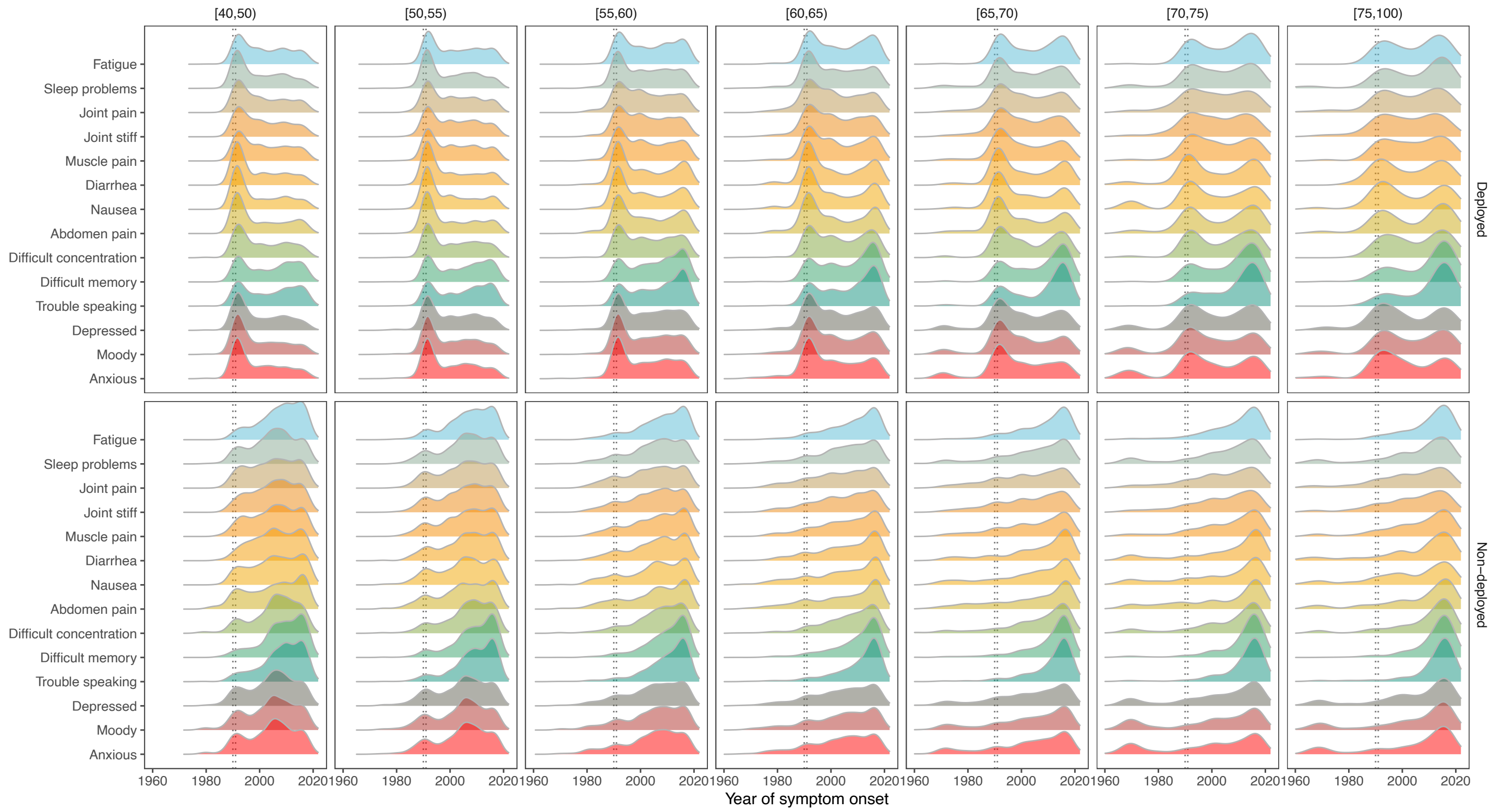

S4. Distribution of symptom onset year in Gulf War deployed and non-deployed Gulf War Era Veterans, stratified by age group. The colored areas represent the density distributions of each symptom estimated using a Gaussian kernel density estimator with the bandwidth set to 0.9 times the minimum of the standard deviation and the interquartile range divided by 1.34 times the sample size to the negative one-fifth power. The two vertical dashed lines mark years 1990 and 1991. Trouble speaking, trouble finding words when speaking.

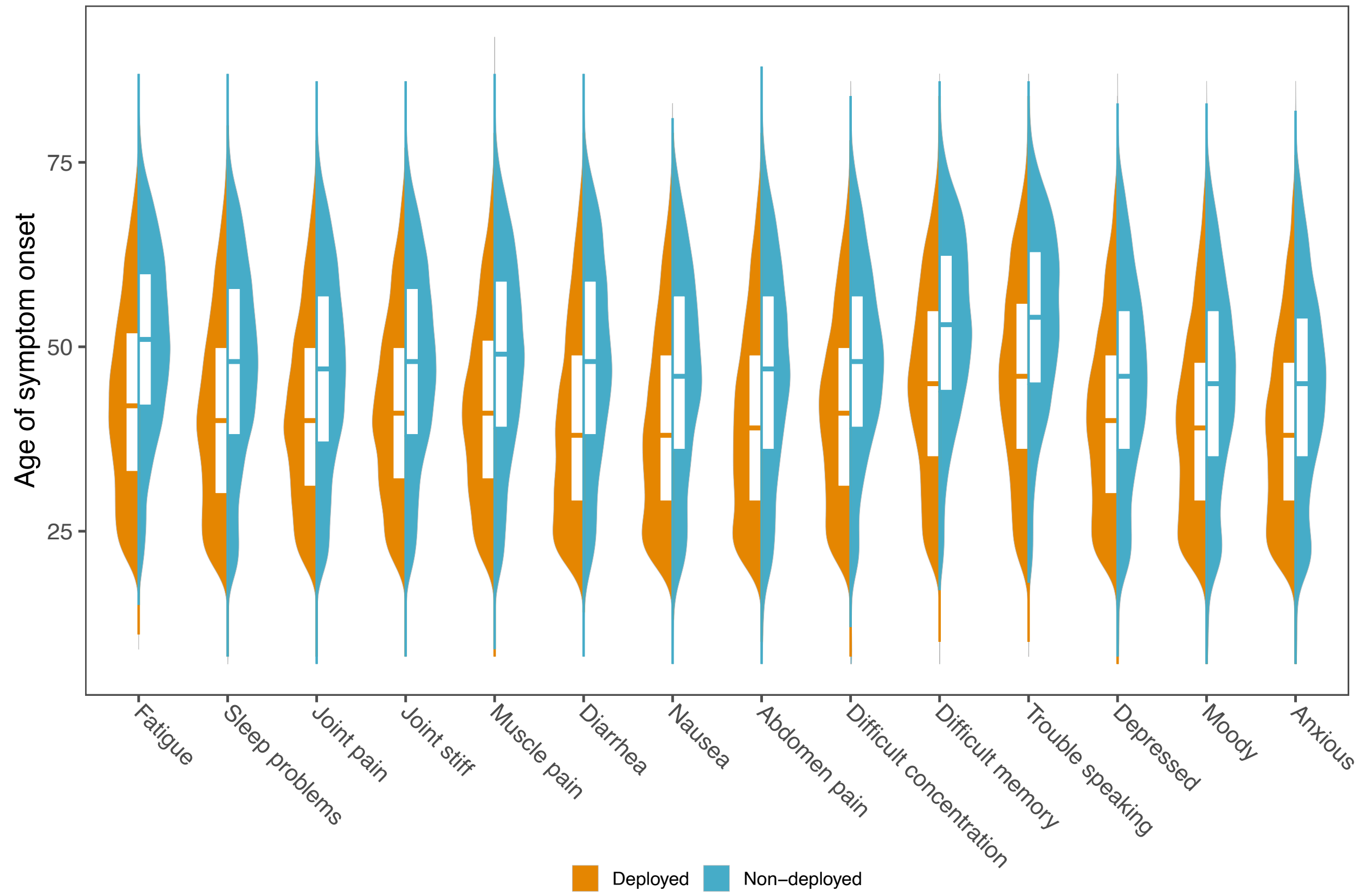

S5. Distribution of onset age for Gulf War Illness symptoms. Trouble speaking, trouble finding words when speaking.

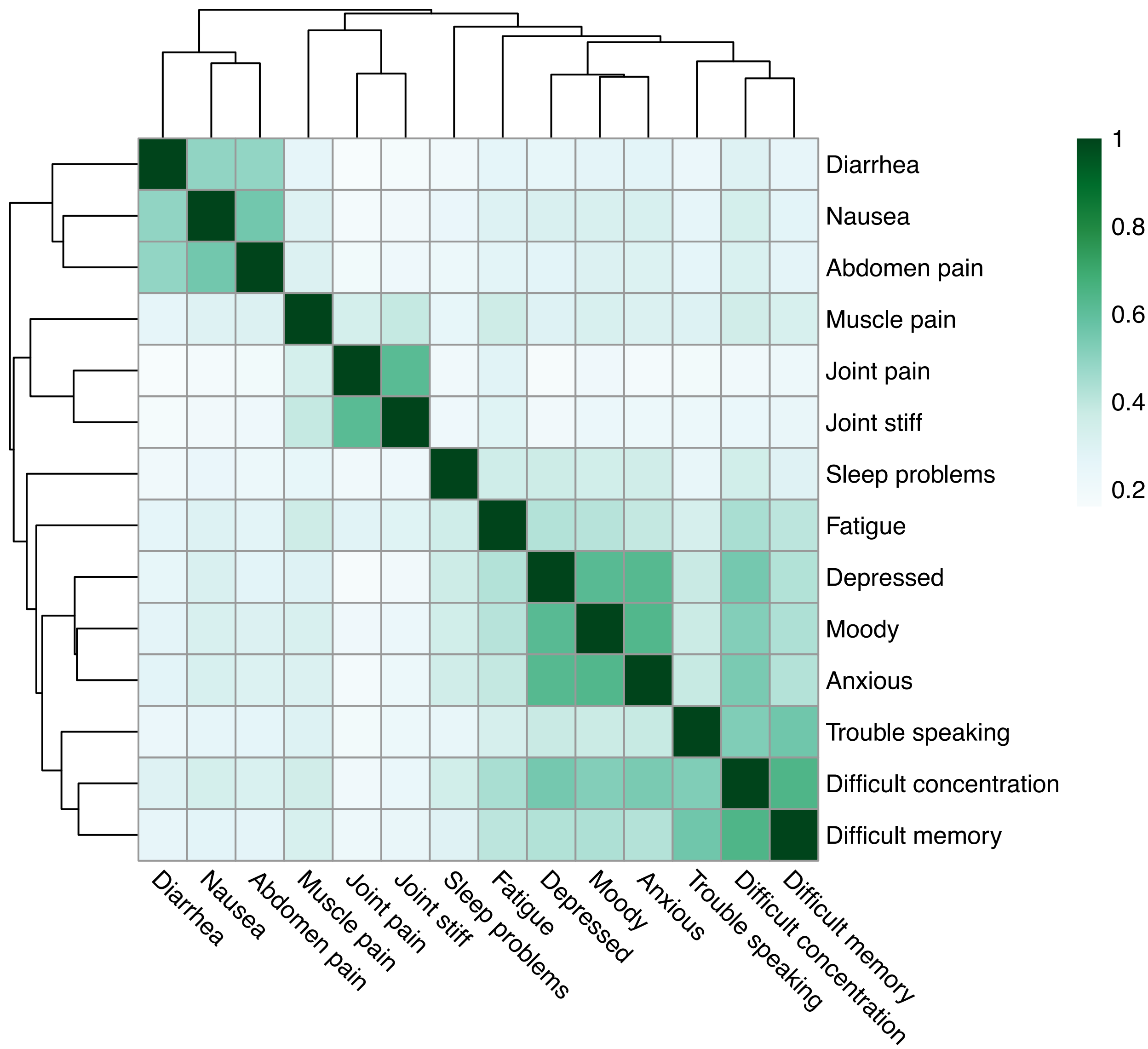

S6. Phenotypic correlations between pairs of Gulf War Illness symptom presence in Gulf War deployed Veterans. Detailed correlation coefficients are presented in Table S5. Trouble speaking, trouble finding words when speaking.

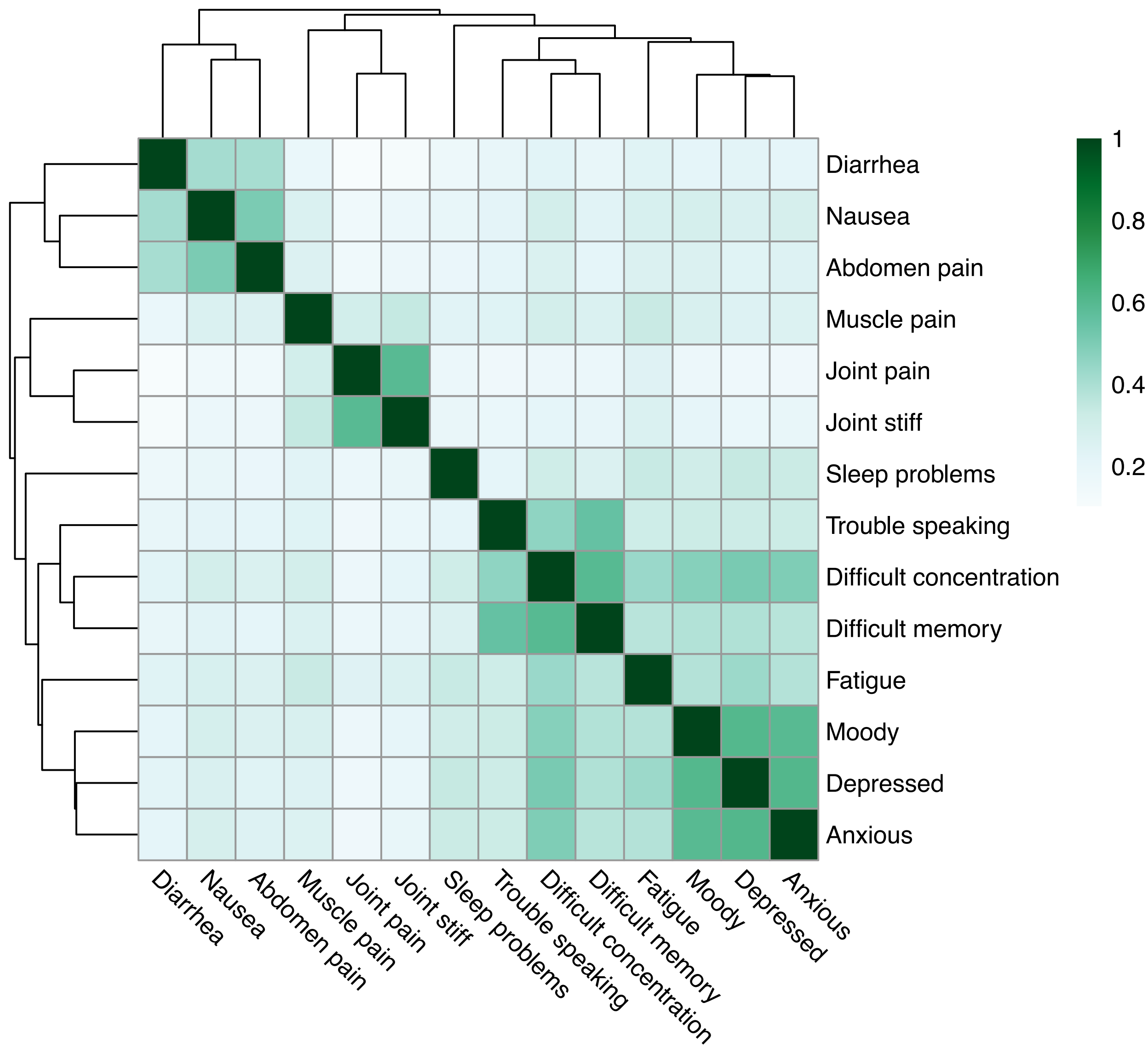

S7. Phenotypic correlations between pairs of Gulf War Illness symptom presence in Gulf War non-deployed Veterans. Detailed correlation coefficients are presented in Table S5. Trouble speaking, trouble finding words when speaking.

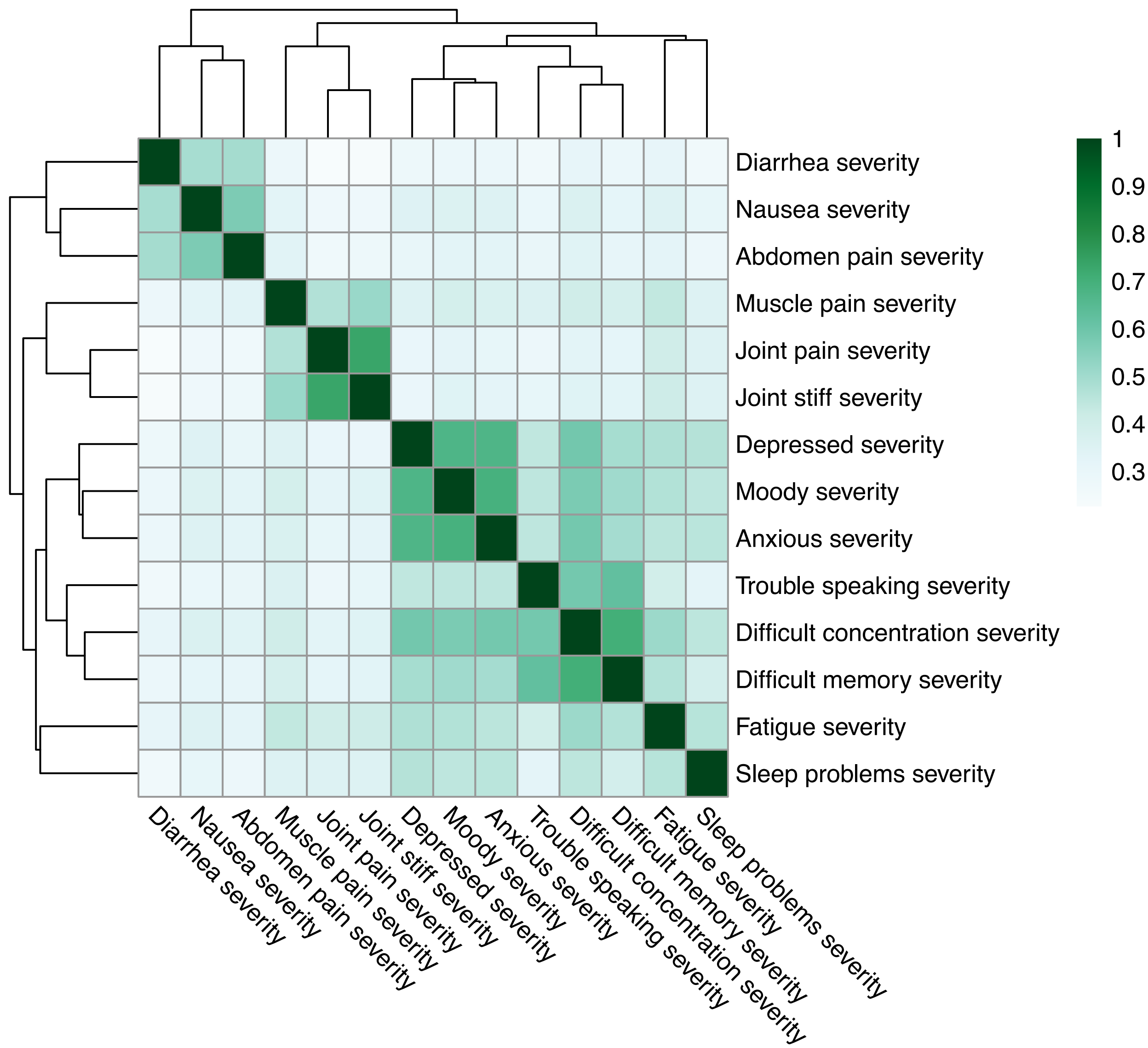

S8. Phenotypic correlations between pairs of Gulf War Illness symptom severity in Gulf War deployed Veterans. Detailed correlation coefficients are presented in Table S5. Trouble speaking, trouble finding words when speaking.

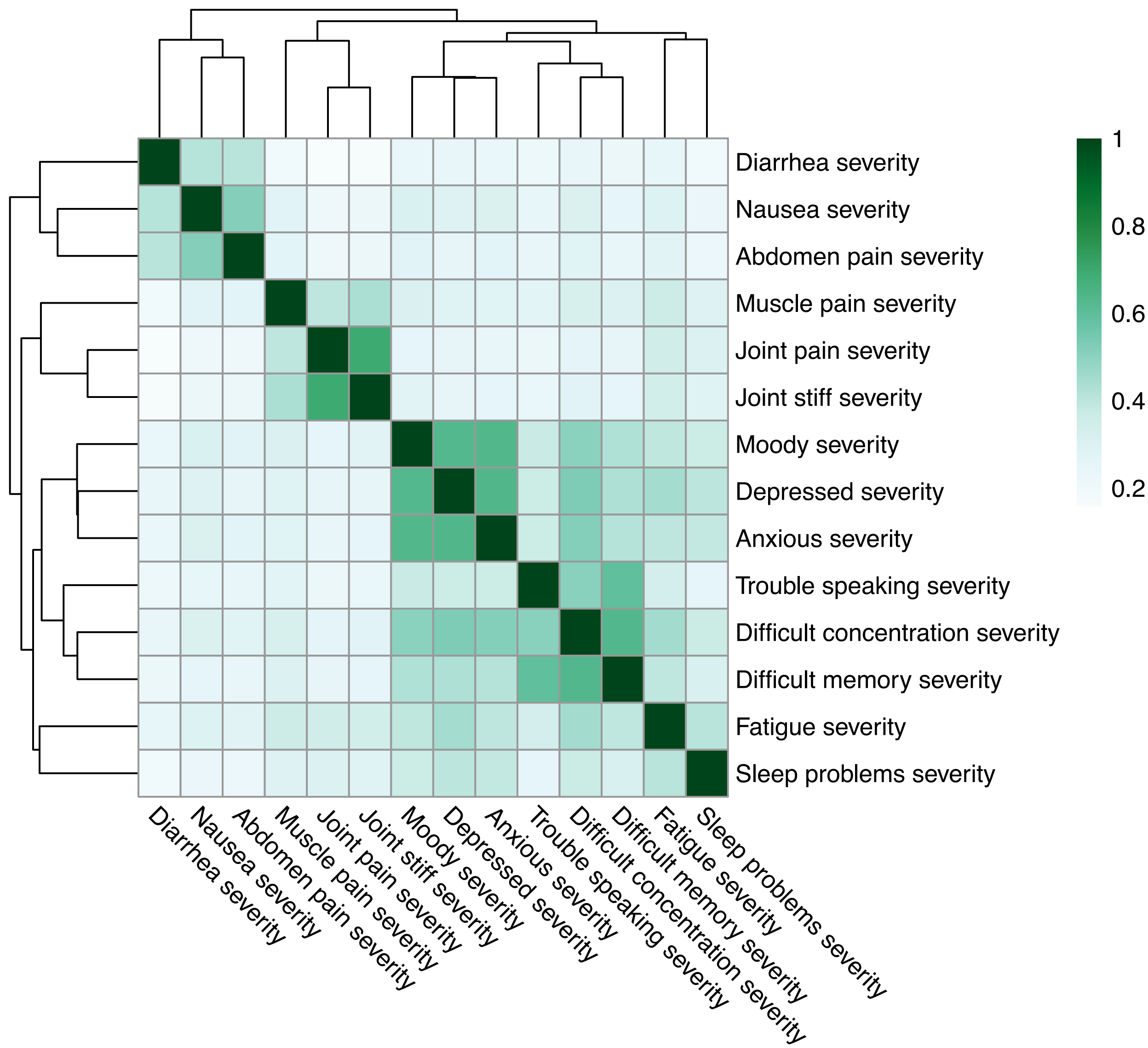

S9. Phenotypic correlations between pairs of Gulf War Illness symptom severity in Gulf War non-deployed Veterans. Detailed correlation coefficients are presented in Table S5. Trouble speaking, trouble finding words when speaking.

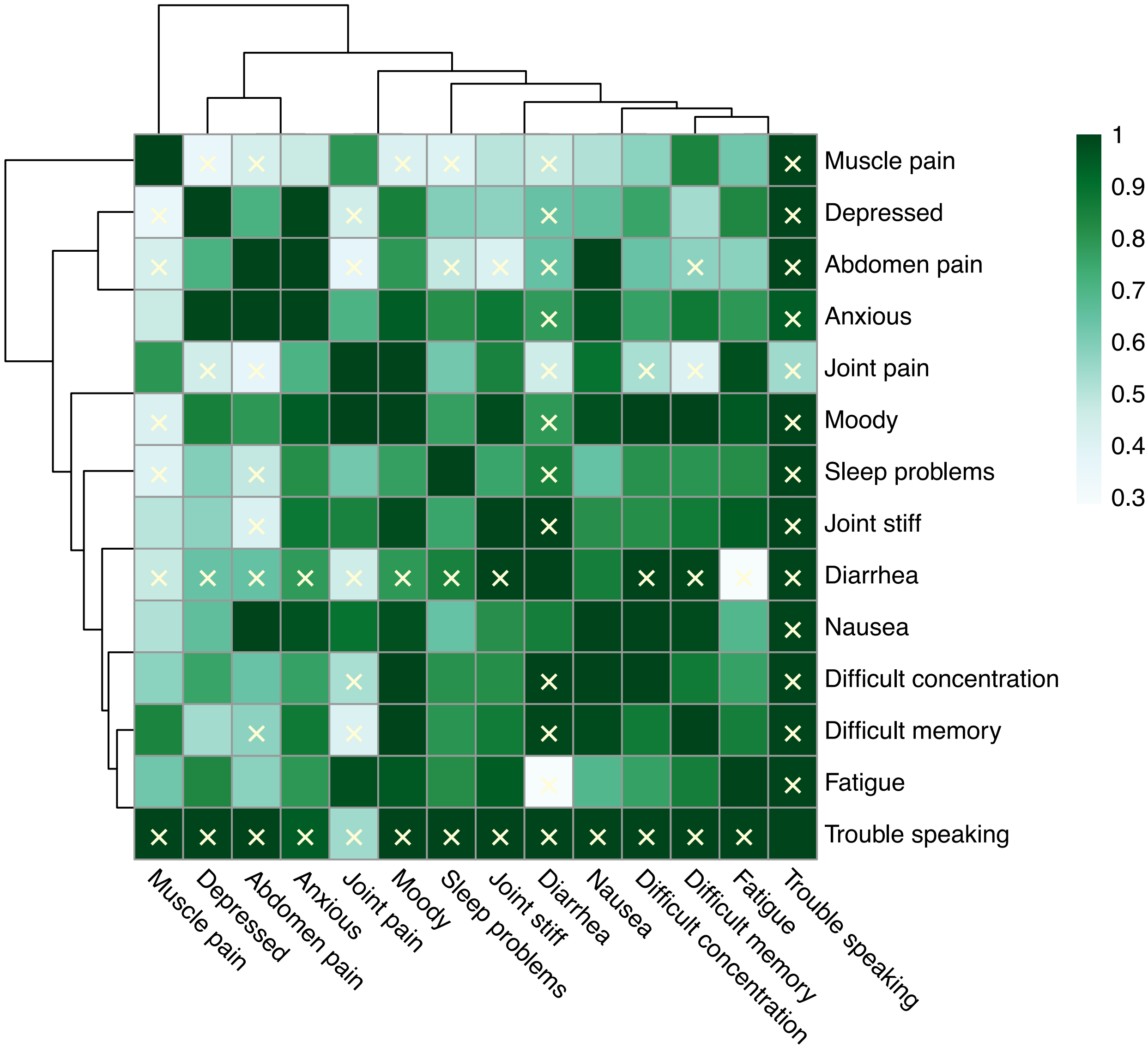

S10. Genetic correlations between pairs of Gulf War Illness symptom presence in Gulf War deployed Veterans. Light yellow crosses indicate nominally non-significant genetic correlations. Detailed correlation coefficients are presented in Table S5. Trouble speaking, trouble finding words when speaking.

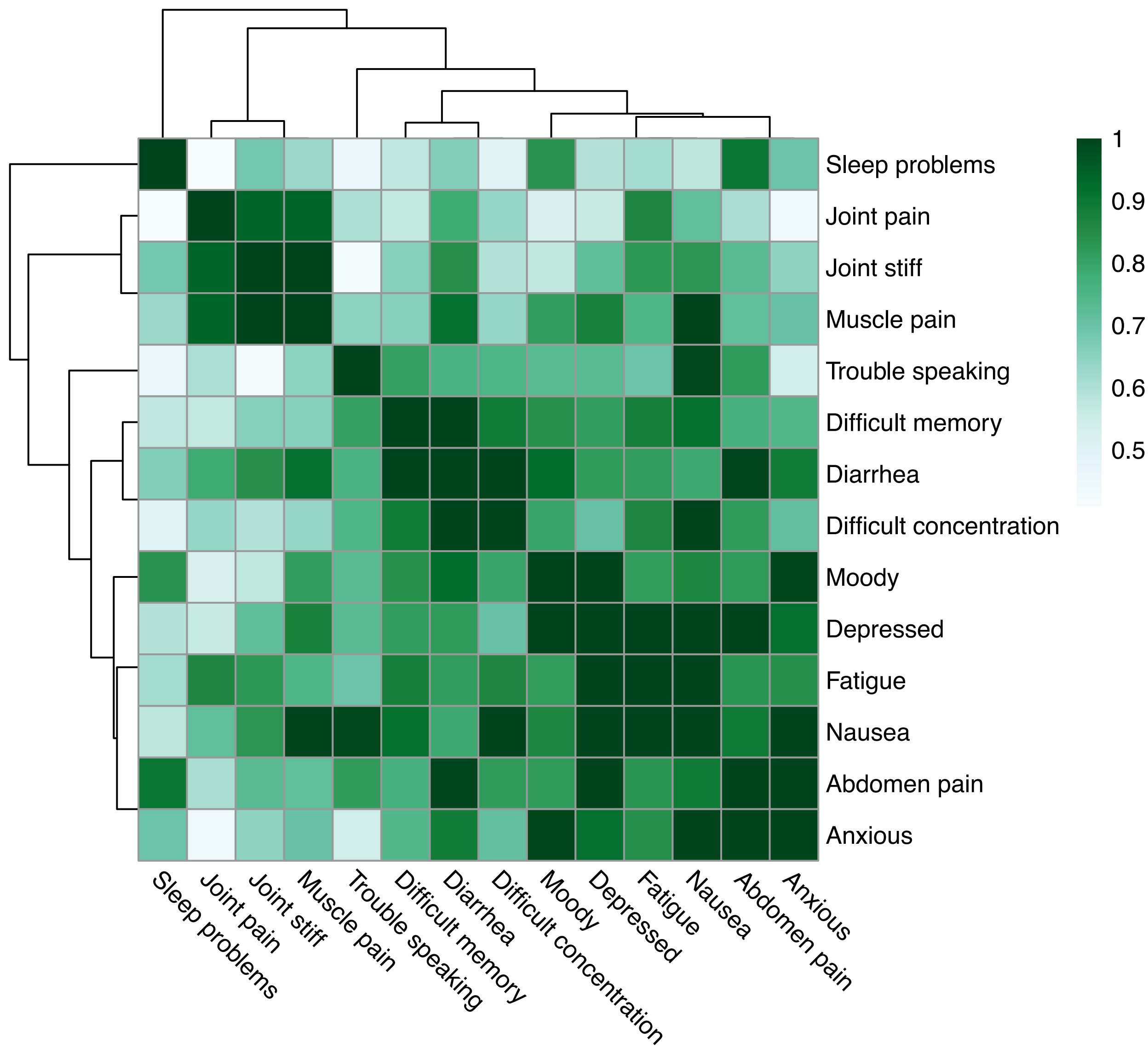

S11. Genetic correlations between pairs of Gulf War Illness symptom presence in Gulf War non-deployed Veterans. Detailed correlation coefficients are presented in Table S5. Trouble speaking, trouble finding words when speaking.

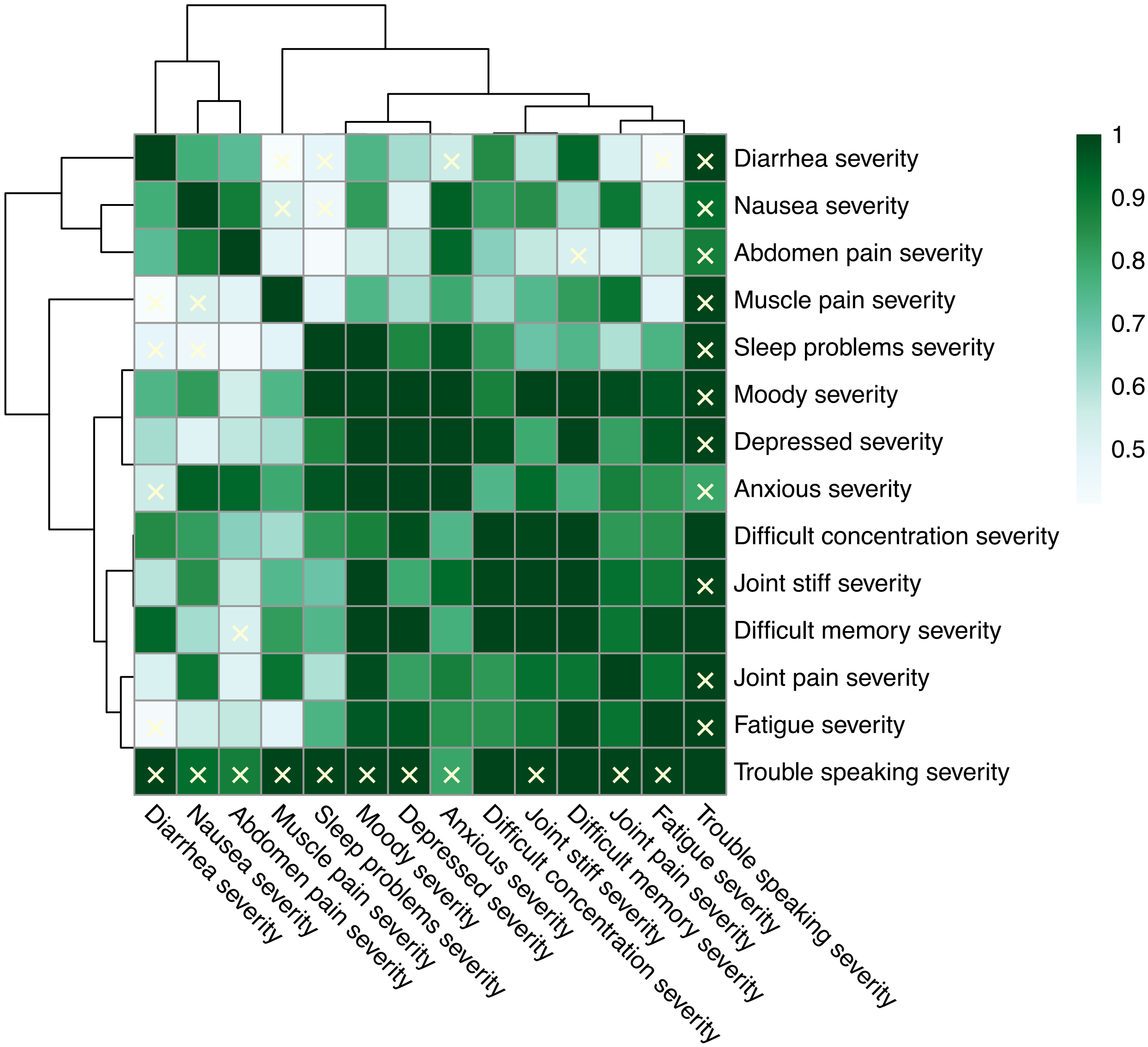

S12. Genetic correlations between pairs of Gulf War Illness symptom severity in Gulf War deployed Veterans. Light yellow crosses indicate nominally non-significant genetic correlations. Detailed correlation coefficients are presented in Table S5. Trouble speaking, trouble finding words when speaking.

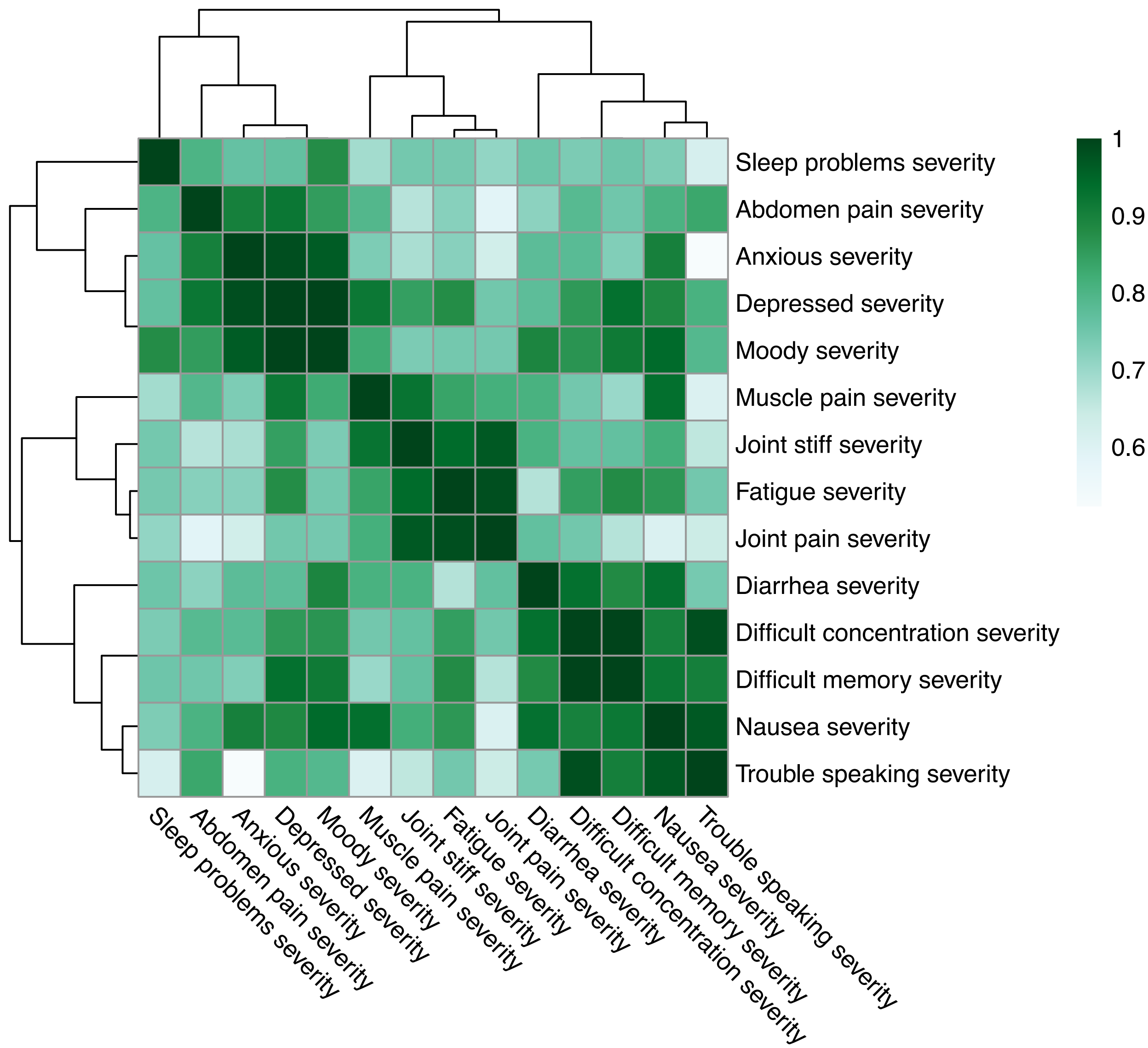

S13. Genetic correlations between pairs of Gulf War Illness symptom severity in Gulf War non-deployed Veterans. Detailed correlation coefficients are presented in Table S5. Trouble speaking, trouble finding words when speaking.

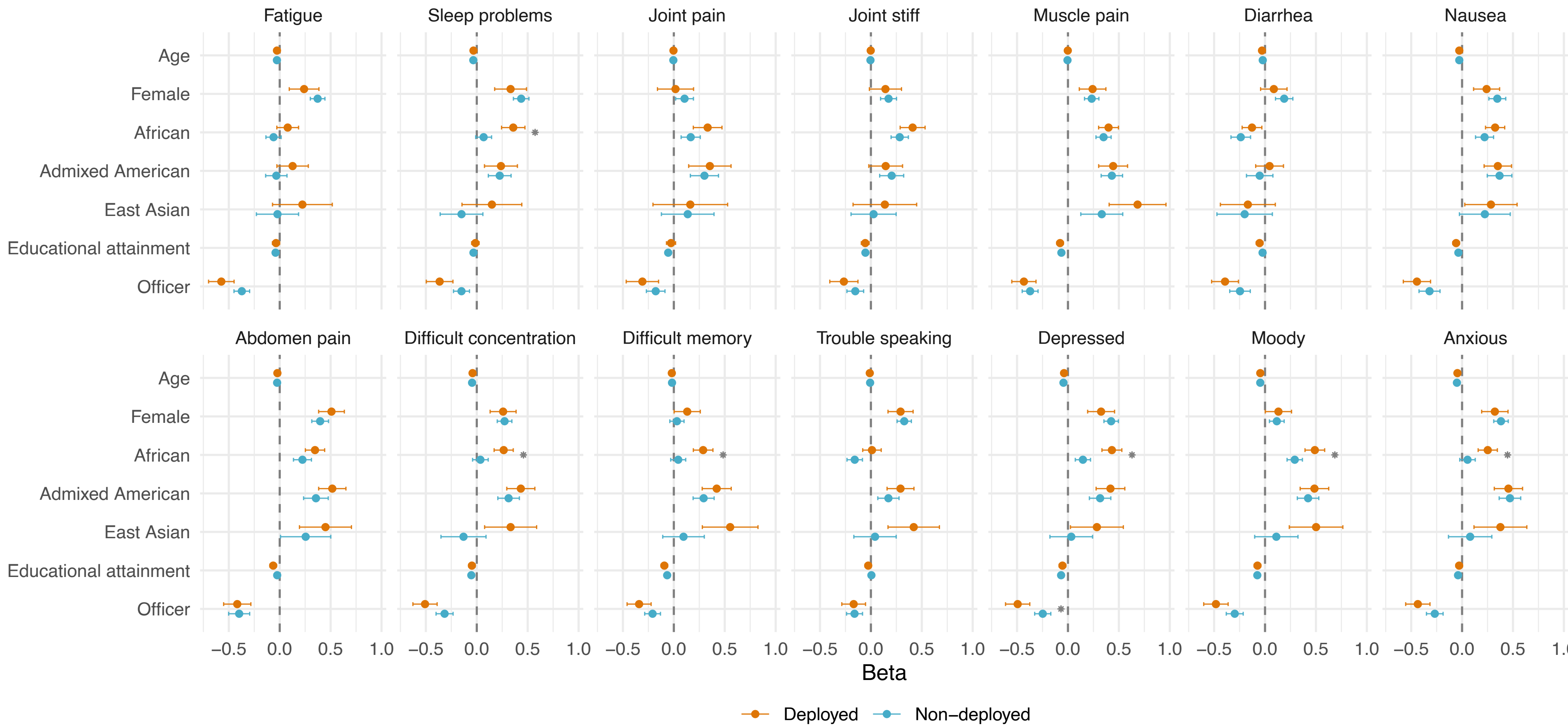

S14. Gulf War deployment specific associations of Gulf War Illness symptom presence with demographics. Multivariable logistic regression models were performed to assess the associations. The reference group for sex, ancestry, and rank were male, European ancestry, and enlisted Veterans, respectively. Star symbols mark statistically significant differences in the effect sizes between Gulf War deployed and non-deployed Veterans surviving Bonferroni multiple testing correction. Trouble speaking, trouble finding words when speaking.

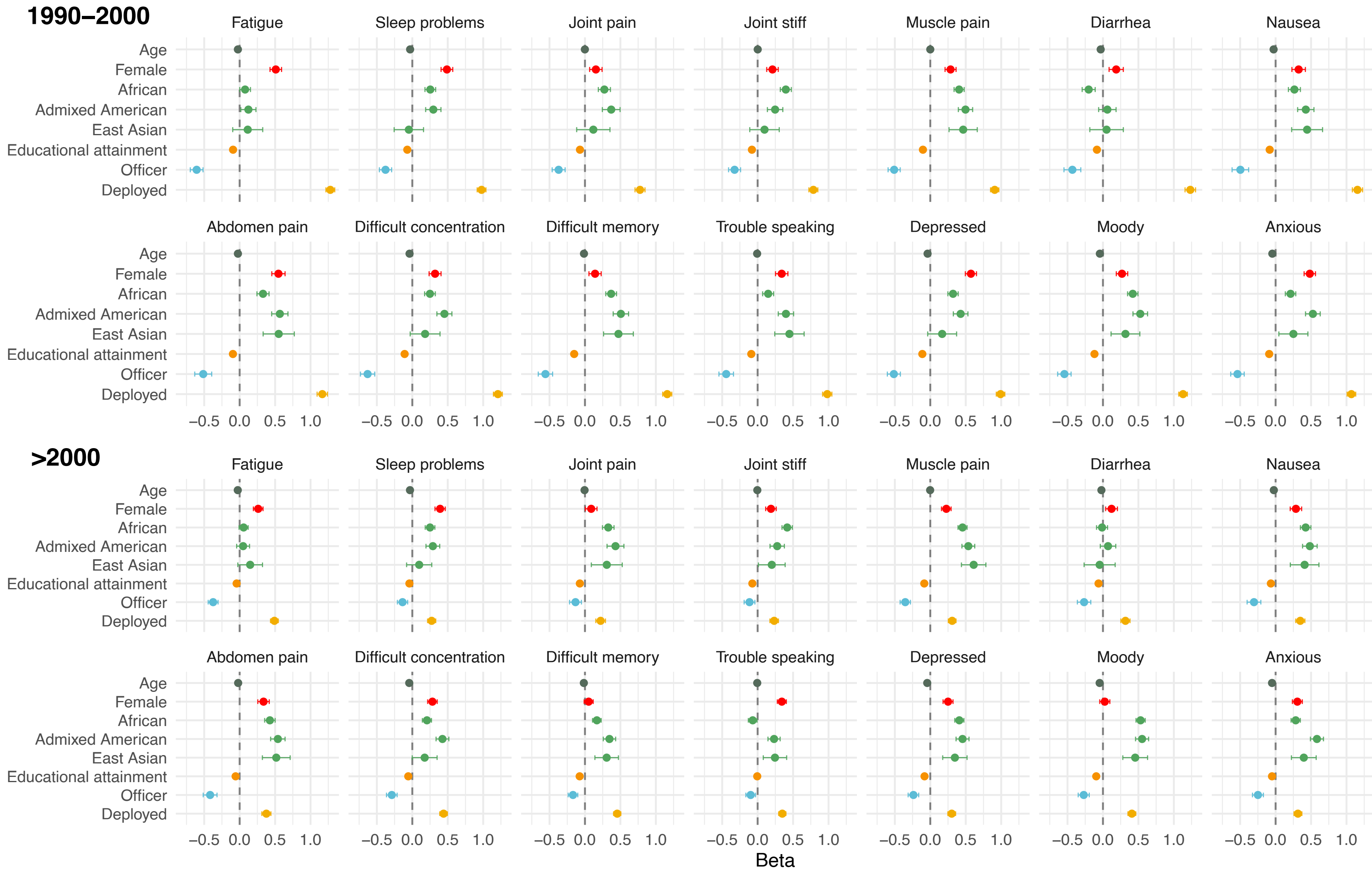

S15. Associations of Gulf War Illness symptom presence with demographics and deployment status stratified by symptom onset year. Multivariable logistic regression models were performed to assess the associations. The reference group for sex, ancestry, rank, and deployment were male, European ancestry, enlisted Veterans, and Gulf War non-deployed Veterans, respectively. Analysis for the upper panel only included cases with symptoms occurred between year 1990 to 2000, while the lower panel only included cases with symptoms occurred after year 2000. Controls were those without the specific symptom in both panels. Trouble speaking, trouble finding words when speaking.

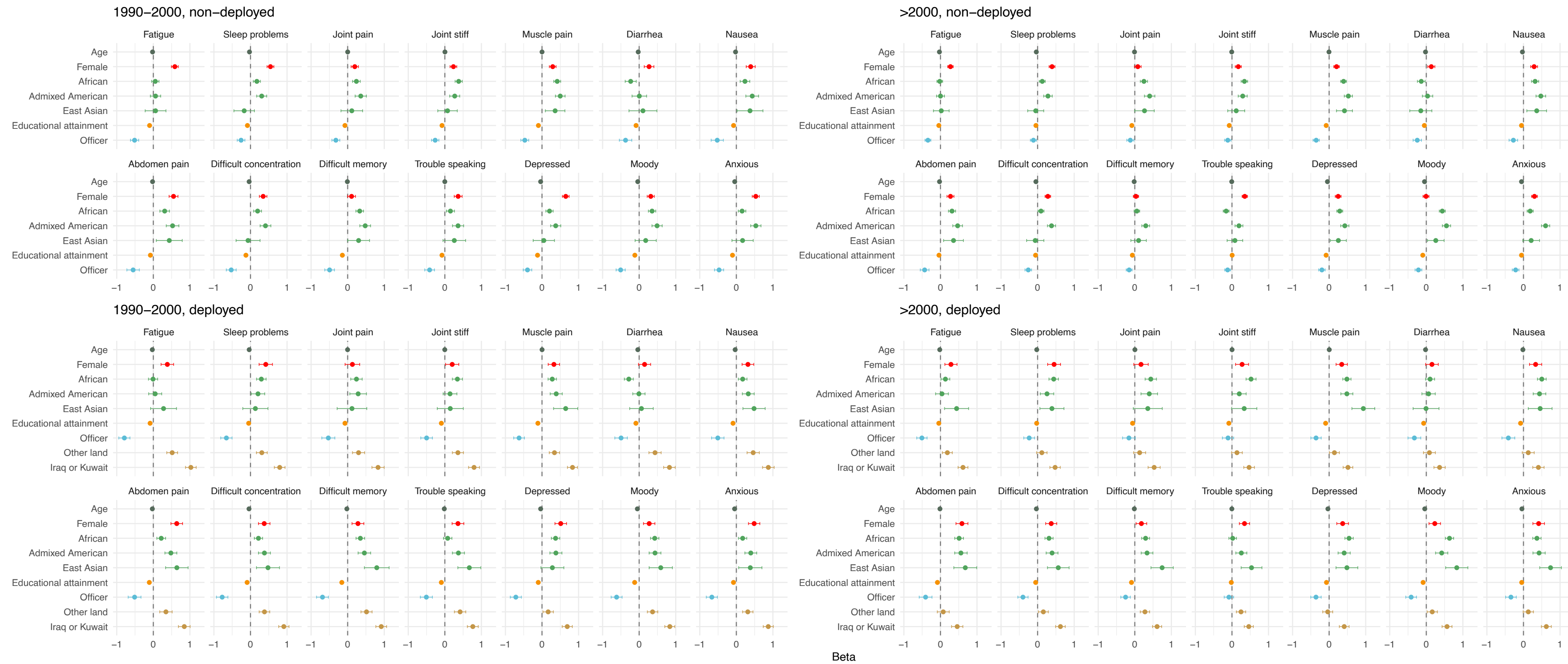

S16. Associations of Gulf War Illness symptom presence with demographics stratified by symptom onset year and deployment status. Multivariable logistic regression models were performed to assess the associations. The reference group for sex, ancestry, and rank were male, European ancestry, and enlisted Veterans, respectively. Analysis for 1990-2000 only included cases with symptoms occurred between year 1990 to 2000, and that for >2000 only included cases with symptoms occurred after year 2000. Controls were those without the specific symptom in each sample. Trouble speaking, trouble finding words when speaking.

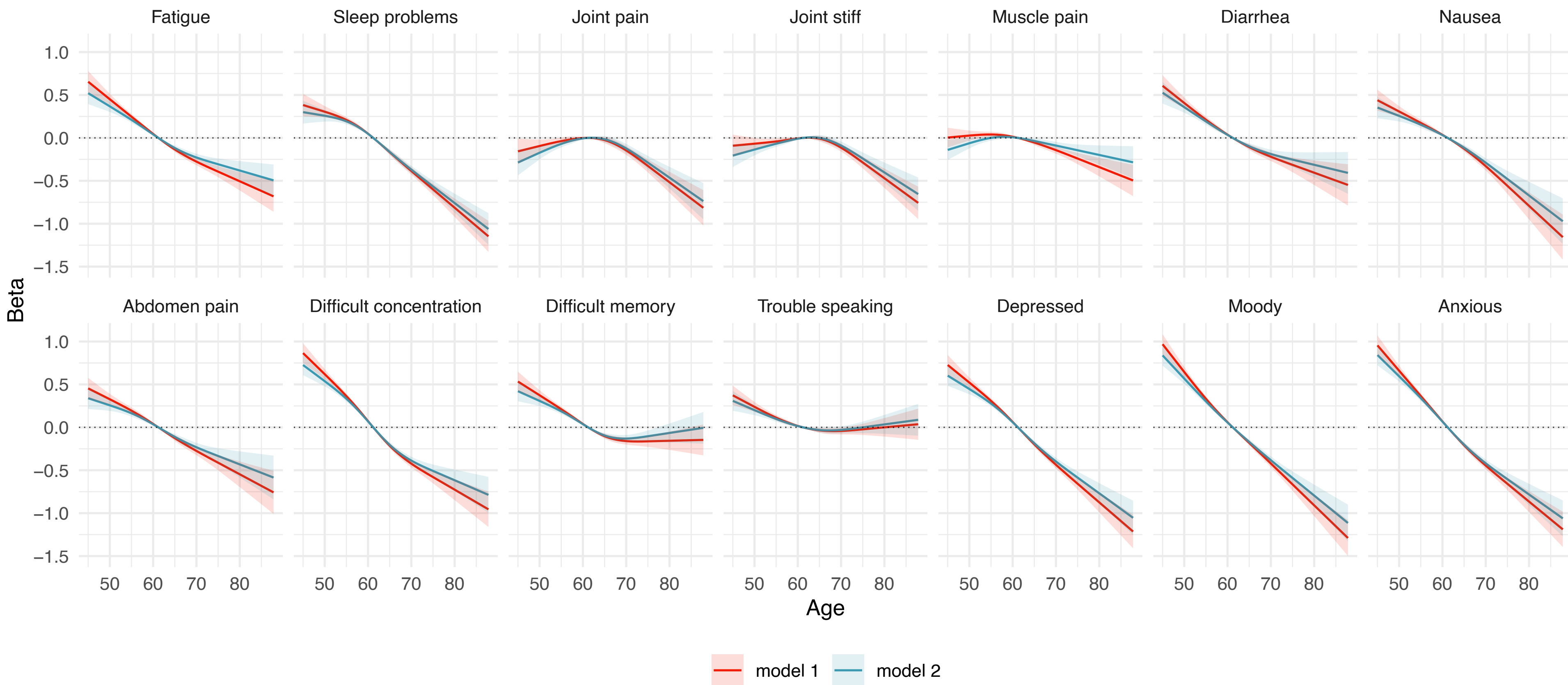

S17. Non-linear associations between age and Gulf War Illness symptom presence in overall Veterans. Restricted cubic spline functions with 5 nodes were incorporated into the regression models to estimate the non-linear associations. Model 1 was adjusted for sex, ancestry, and deployment status; Model 2 was further adjusted for educational attainment, military rank, and service branch. Trouble speaking, trouble finding words when speaking.

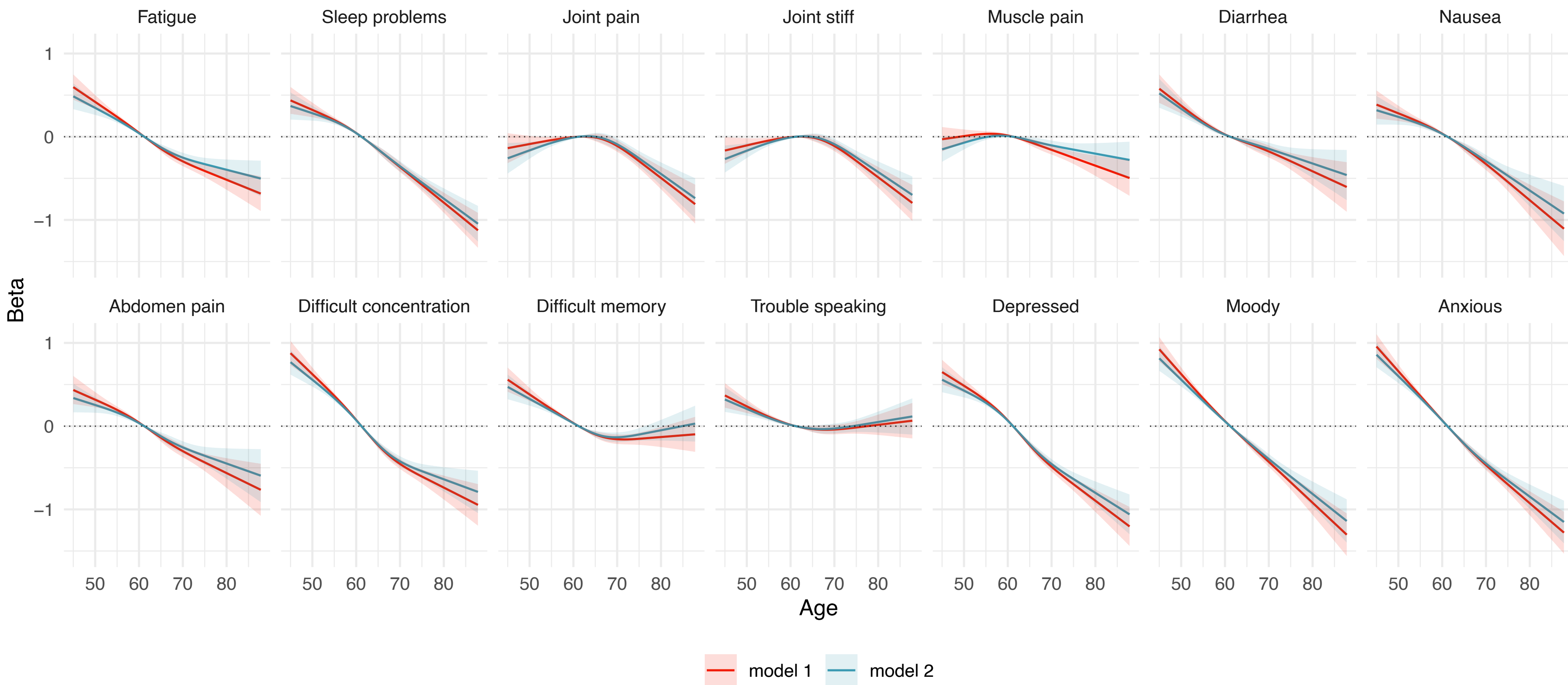

S18. Non-linear associations between age and Gulf War Illness symptom presence in Gulf War non-deployed Veterans. Restricted cubic spline functions with 5 nodes were incorporated into the regression models to estimate the non-linear associations. Model 1 was adjusted for sex and ancestry; Model 2 was further adjusted for educational attainment, military rank, and service branch. Trouble speaking, trouble finding words when speaking.

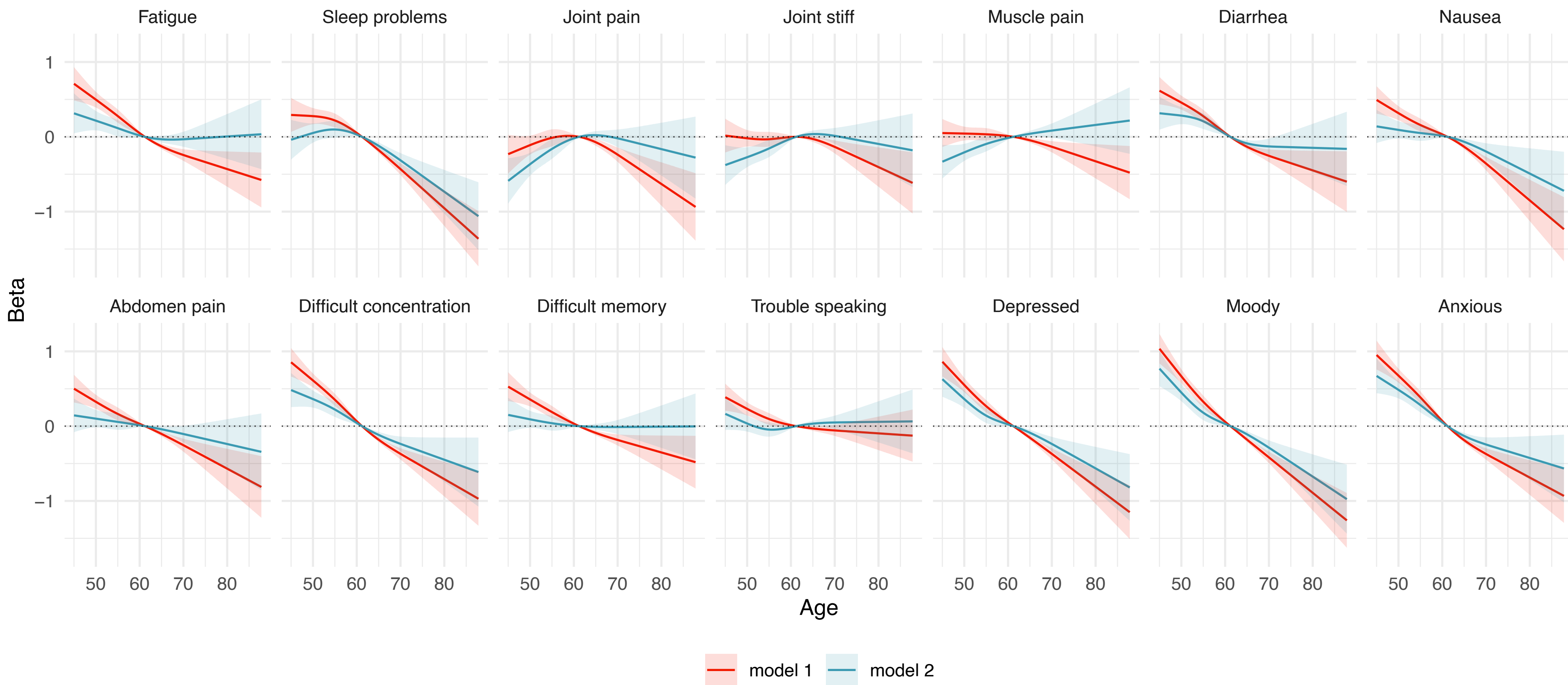

S19. Non-linear associations between age and Gulf War Illness symptom presence in Gulf War deployed Veterans. Restricted cubic spline functions with 5 nodes were incorporated into the regression models to estimate the non-linear associations. Model 1 was adjusted for sex and ancestry; Model 2 was further adjusted for educational attainment, military rank, service branch, and eight GW-related environmental exposures (i.e., smoke from oil well fires, ground combat, anti-nerve agent, chemical or biological warfare agents, prisoners, pesticide cream, pesticides-treated uniform, and insect baits). Trouble speaking, trouble finding words when speaking.

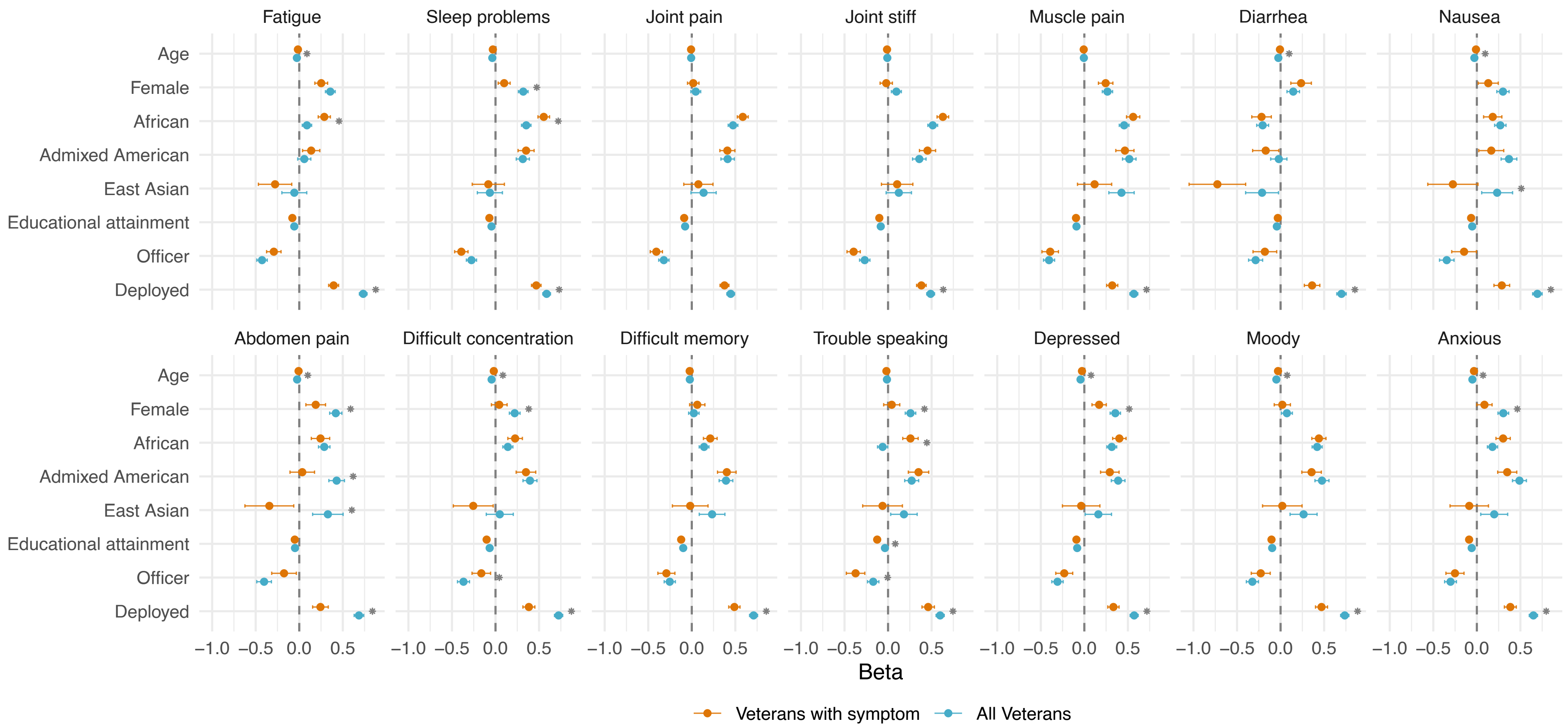

S20. Associations of Gulf War Illness symptom severity with demographics and Gulf War deployment status in all Veterans and Veterans with a specific symptom. Multivariable ordinal logistic regression models were performed to assess the associations. The reference group for sex, ancestry, rank and Gulf War deployment status were male, European ancestry, enlisted Veterans and Gulf War non-deployed Veterans, respectively. Star symbols mark statistically significant differences in the effect sizes between the two samples surviving Bonferroni multiple testing correction. Trouble speaking, trouble finding words when speaking.

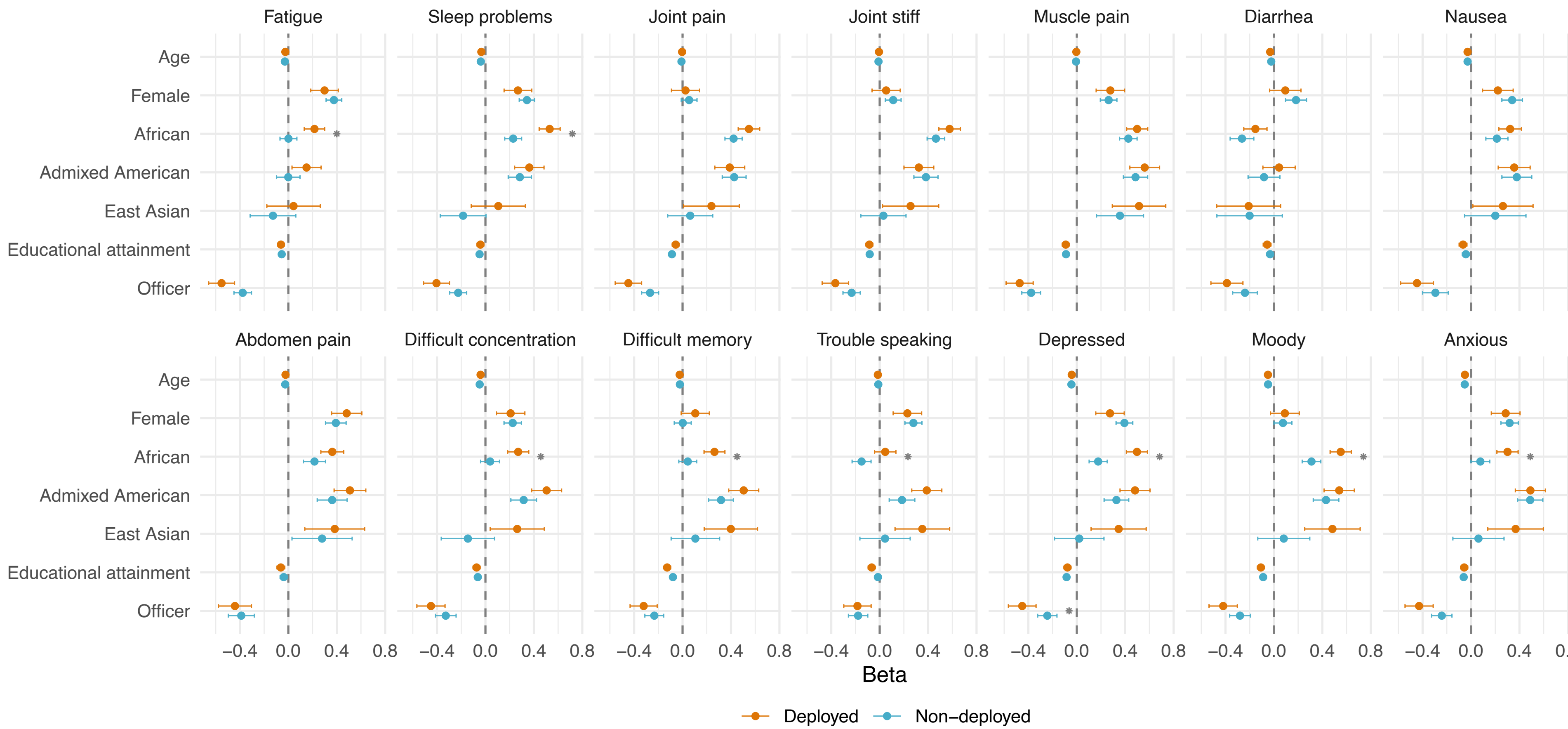

S21. Gulf War deployment specific associations of Gulf War Illness symptom severity with demographics. Multivariable ordinal logistic regression models were performed to assess the associations. The reference group for sex, ancestry, and rank were male, European ancestry, and enlisted Veterans, respectively. Star symbols mark statistically significant differences in the effect sizes between Gulf War deployed and non-deployed Veterans surviving Bonferroni multiple testing correction. Trouble speaking, trouble finding words when speaking.

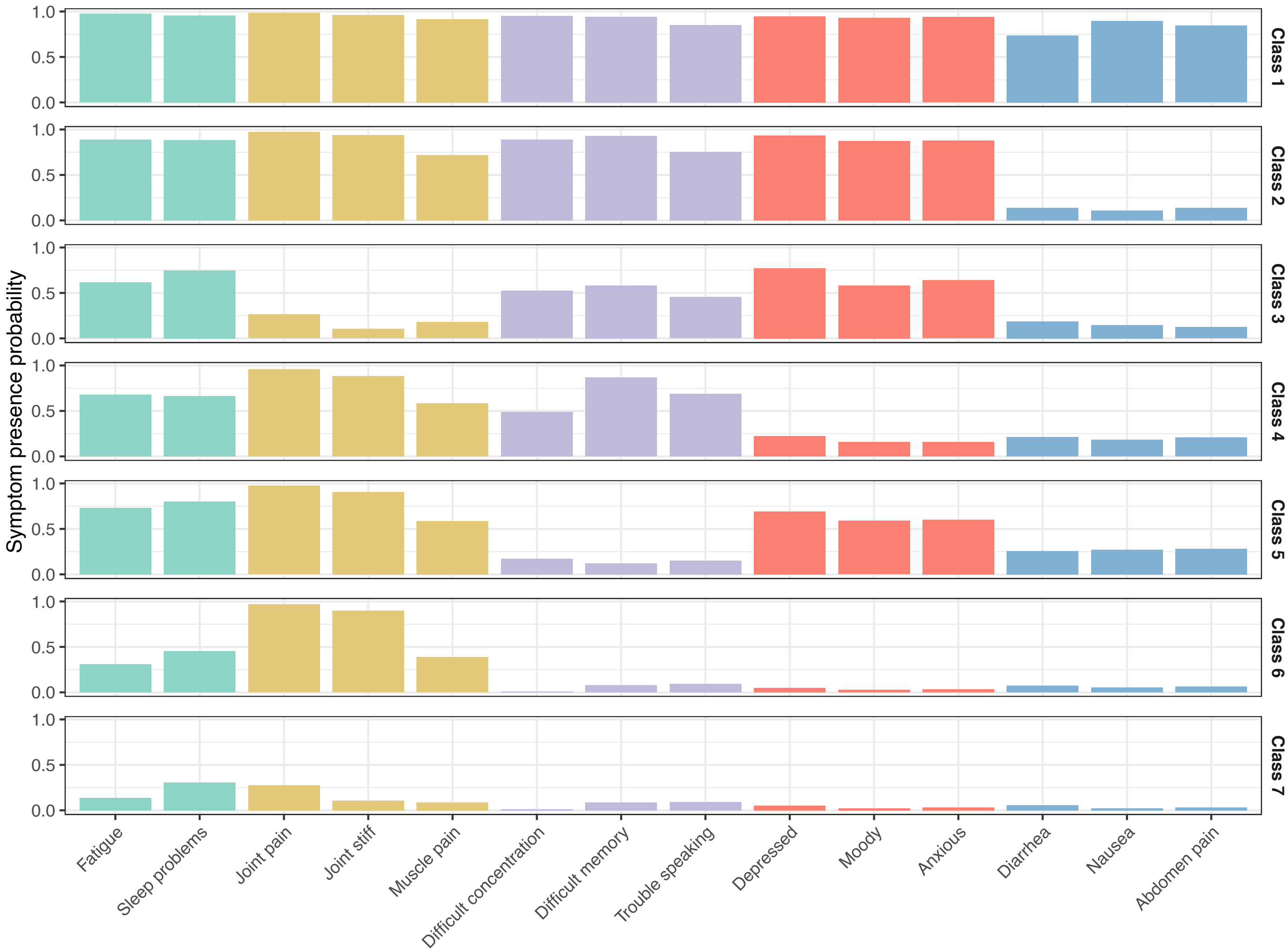

S22. Latent symptom classes in all Veterans identified by latent class analysis based on the presence of 14 Gulf War Illness symptoms. Trouble speaking, trouble finding words when speaking.

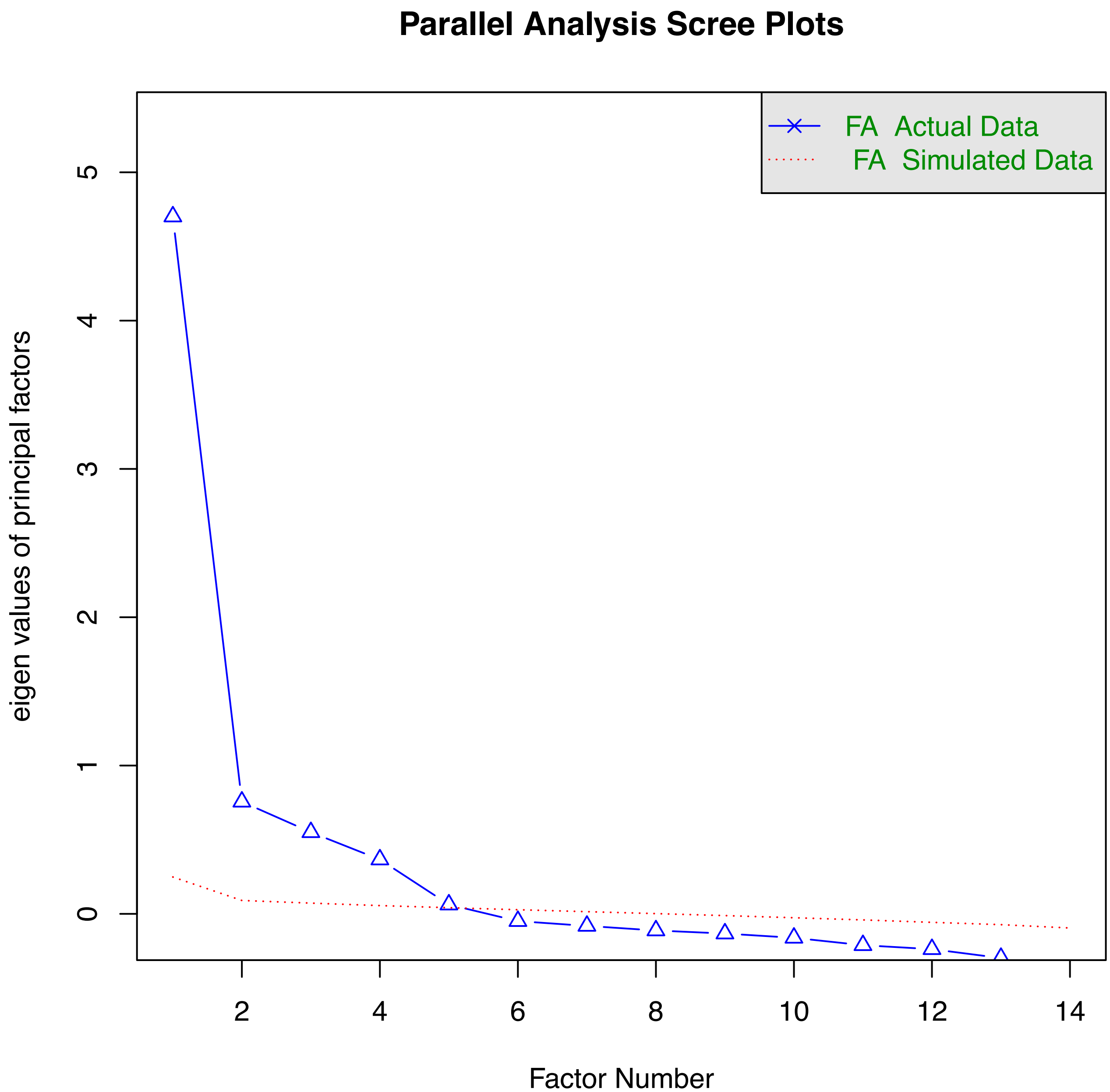

S23. Parallel analysis scree plot for Gulf War Illness symptom presence in Gulf War deployed Veterans.

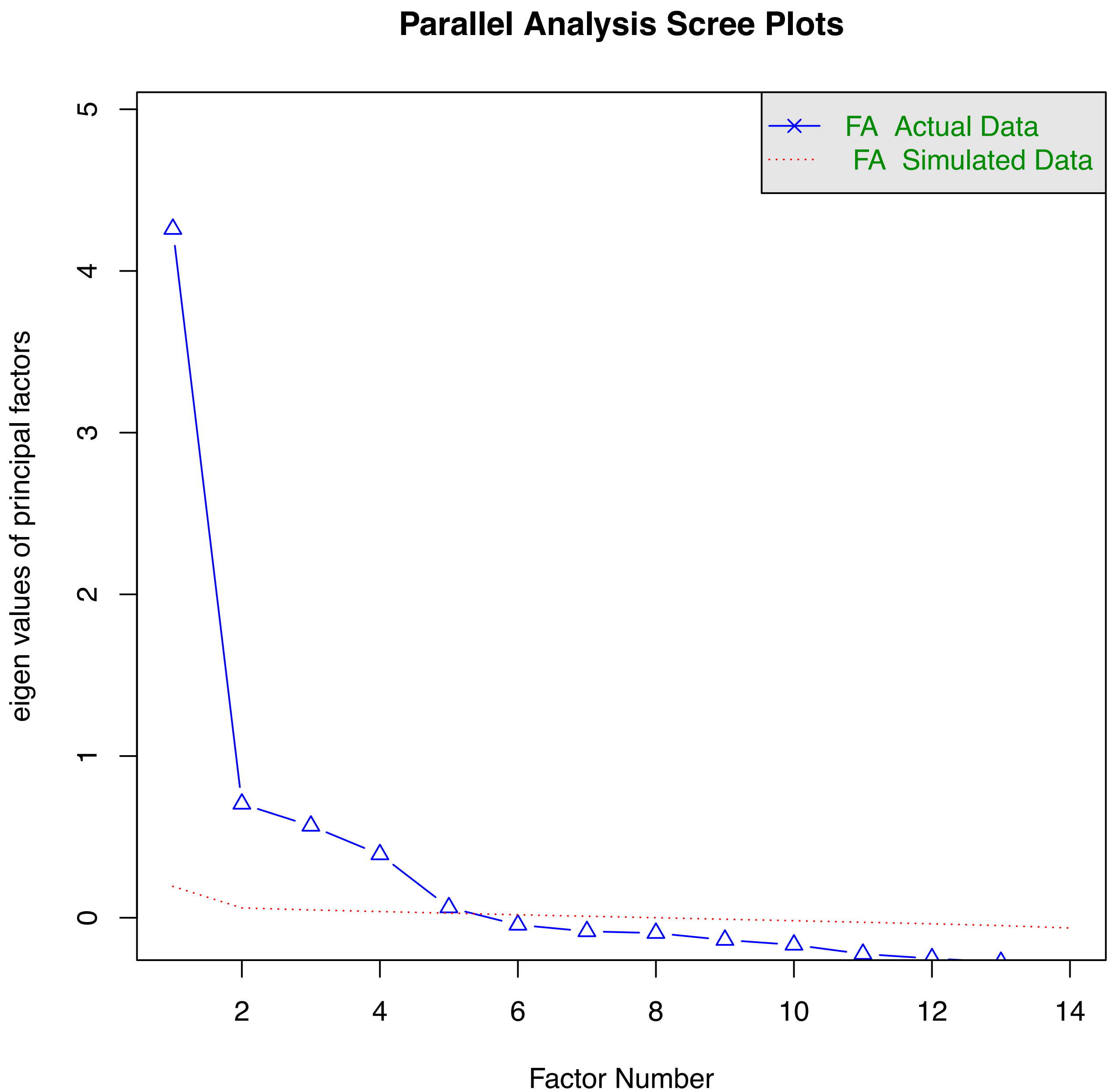

S24. Parallel analysis scree plot for Gulf War Illness symptom presence in Gulf War non-deployed Veterans.

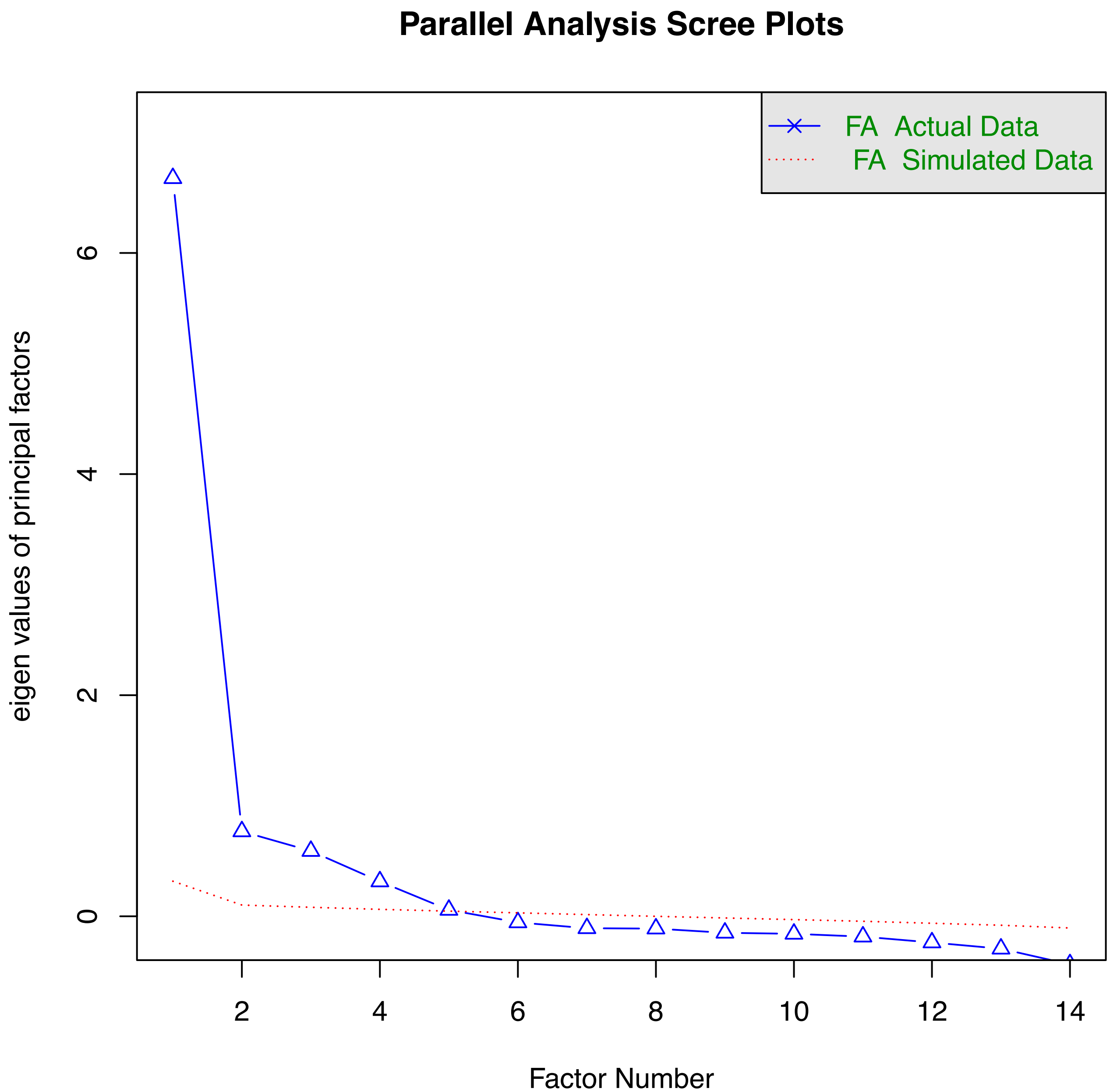

S25. Parallel analysis scree plot for Gulf War Illness symptom severity in Gulf War deployed Veterans.

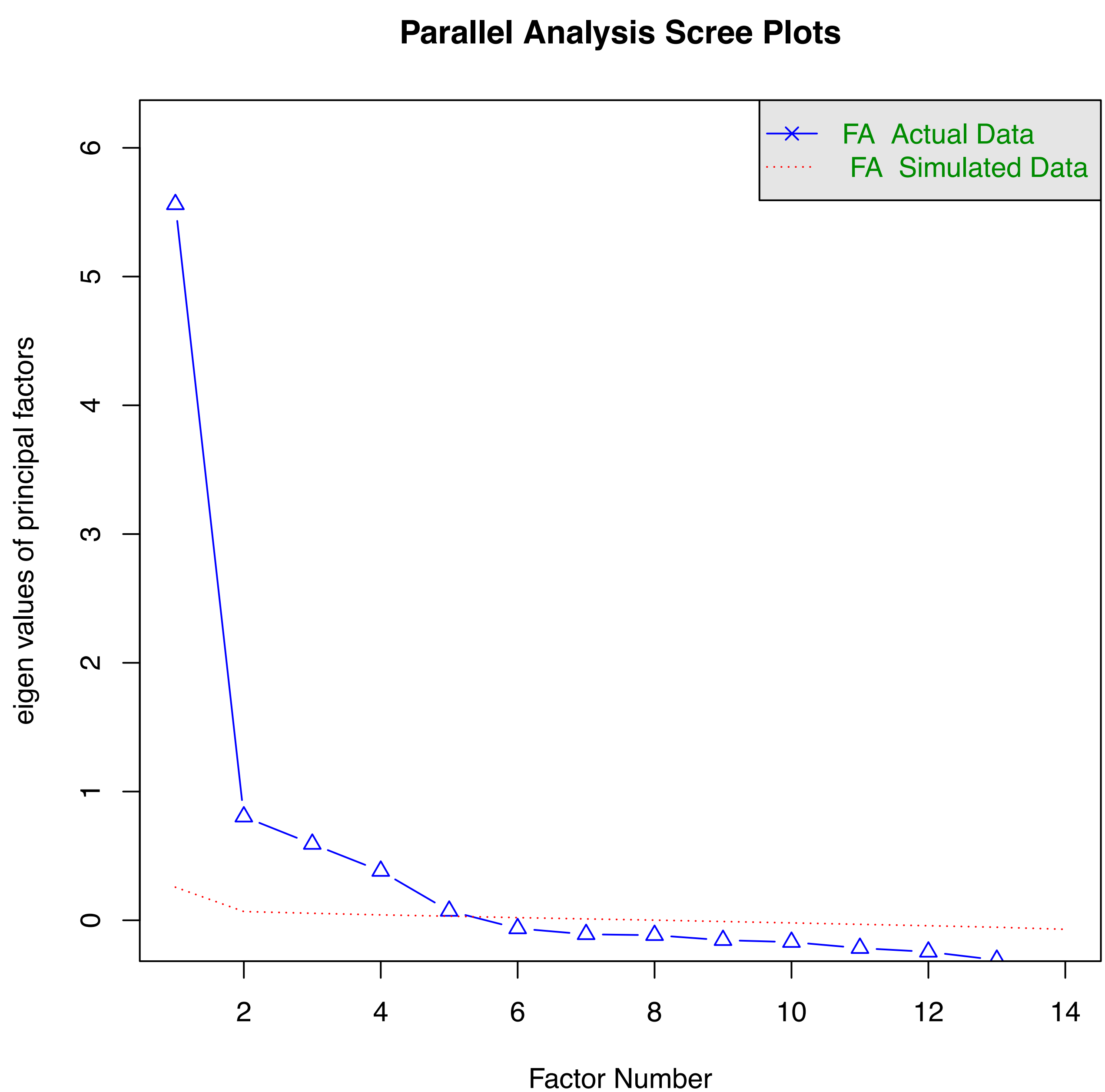

S26. Parallel analysis scree plot for Gulf War Illness symptom severity in Gulf War non-deployed Veterans.

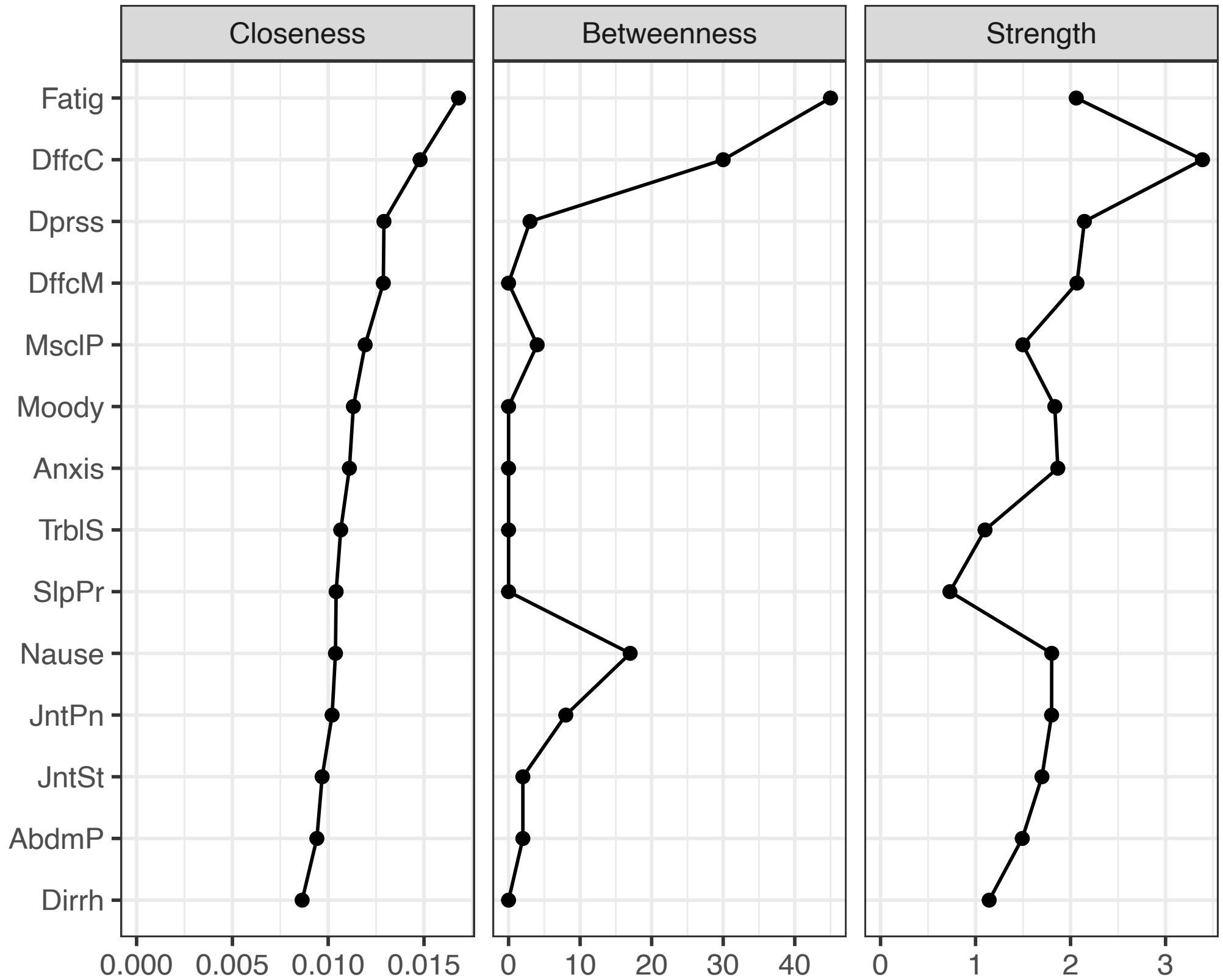

S27. Centrality indices for the network of Gulf War Illness symptom presence in Gulf War deployed Veterans. Abbreviations: Fatig, fatigue; SlpPr, sleep problems; JntPn, joint pain; JntSt, joint stiff; MsclP, muscle pain; Dirrh, diarrhea; Nause, nausea; AbdmP, abdomen pain; DffcC, difficult concentration; DffcM, difficult memory; TrblS, trouble finding words when speaking; Dprss, depressed; Anxis, anxious.

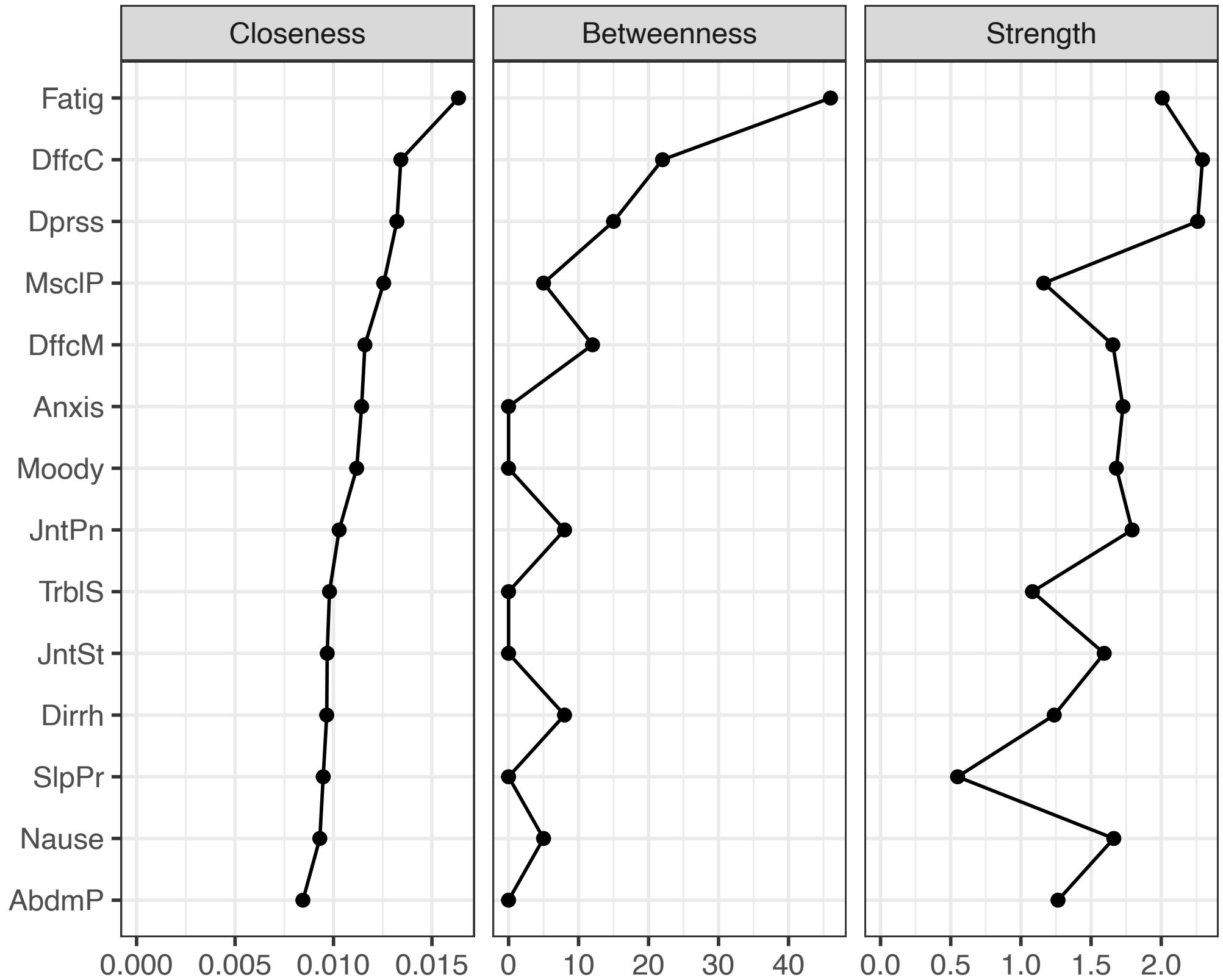

S28. Centrality indices for the network of Gulf War Illness symptom presence in Gulf War non-deployed Veterans. Abbreviations: Fatig, fatigue; SlpPr, sleep problems; JntPn, joint pain; JntSt, joint stiff; MsclP, muscle pain; Dirrh, diarrhea; Nause, nausea; AbdmP, abdomen pain; DffcC, difficult concentration; DffcM, difficult memory; TrblS, trouble finding words when speaking; Dprss, depressed; Anxis, anxious.

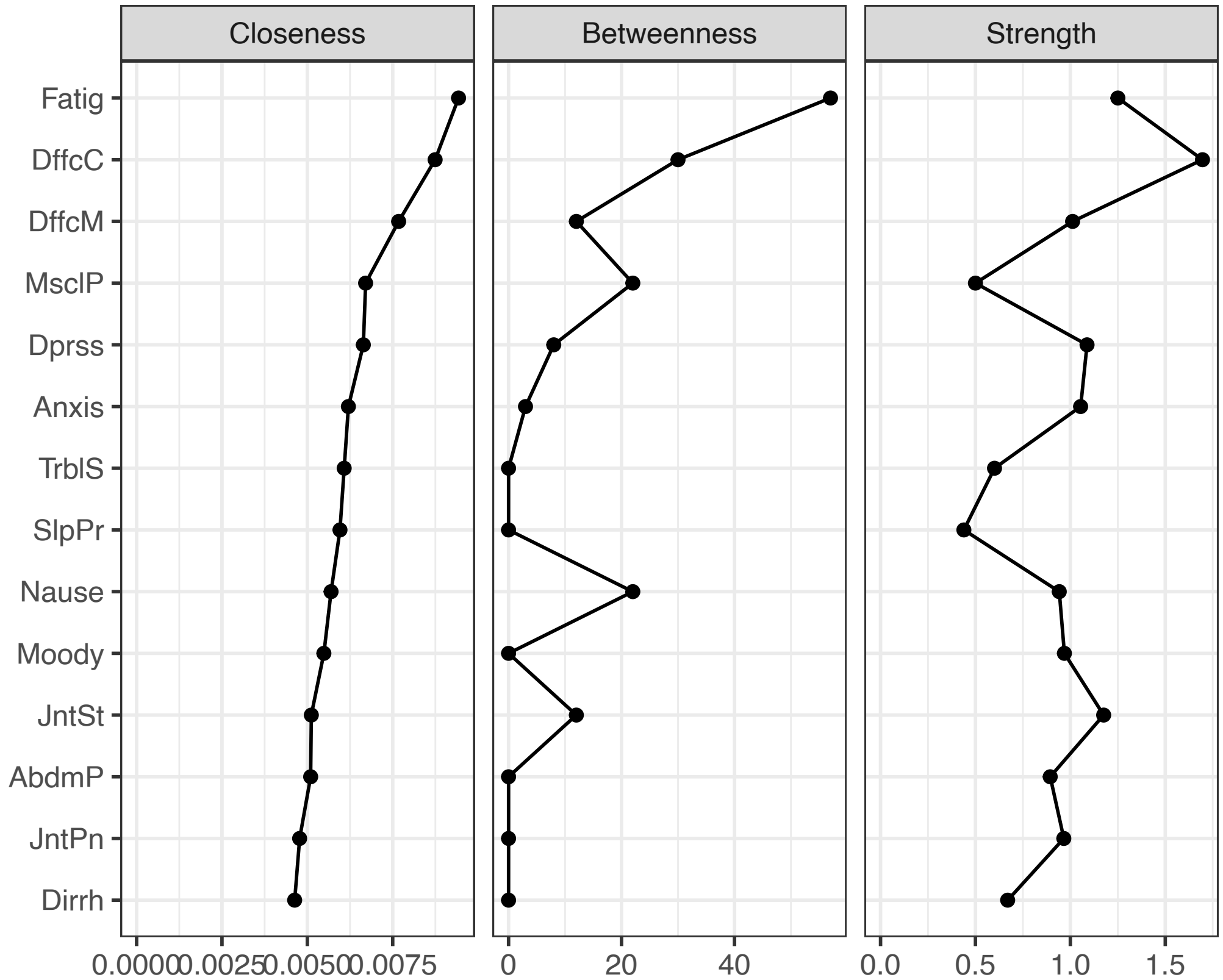

S29. Centrality indices for the network of Gulf War Illness symptom severity in Gulf War deployed Veterans. Abbreviations: Fatig, fatigue; SlpPr, sleep problems; JntPn, joint pain; JntSt, joint stiff; MsclP, muscle pain; Dirrh, diarrhea; Nause, nausea; AbdmP, abdomen pain; DffcC, difficult concentration; DffcM, difficult memory; TrblS, trouble finding words when speaking; Dprss, depressed; Anxis, anxious.

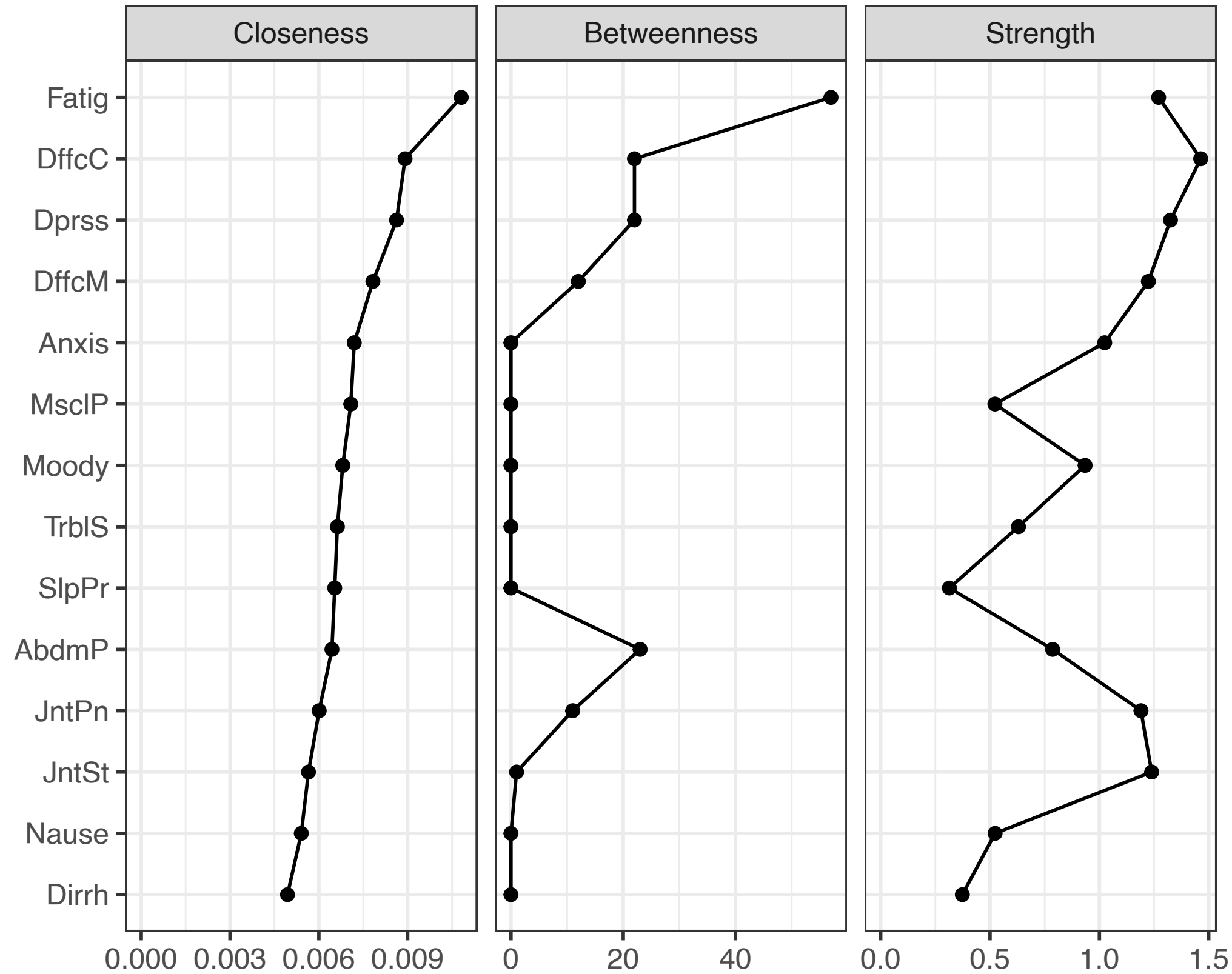

S30. Centrality indices for the network of Gulf War Illness symptom severity in Gulf War non-deployed Veterans. Abbreviations: Fatig, fatigue; SlpPr, sleep problems; JntPn, joint pain; JntSt, joint stiff; MsclP, muscle pain; Dirrh, diarrhea; Nause, nausea; AbdmP, abdomen pain; DffcC, difficult concentration; DffcM, difficult memory; TrblS, trouble finding words when speaking; Dprss, depressed; Anxis, anxious.
